# Directional divergence of Ep300 duplicates in teleosts and its implications

**DOI:** 10.1186/s12862-020-01712-6

**Published:** 2020-10-31

**Authors:** Xianzong Wang, Junli Yan

**Affiliations:** 1 College of Animal Science, Shanxi Agricultural University, Taigu, 030801 China; 2 College of Urban and Rural Construction, Shanxi Agricultural University, Taigu, 030801 China

**Keywords:** EP300, Lysine acetyltransferase, Teleost, Whole genome duplication, Divergent evolution, Positive selection, Radiation

## Abstract

**Background:**

EP300 is a conserved protein in vertebrates, which serves as a key mediator of cellular homeostasis. Mutations and dysregulation of EP300 give rise to severe human developmental disorders and malignancy. *Danio rerio* is a promising model organism to study EP300 related diseases and drugs; however, the effect of EP300 duplicates derived from teleost-specific whole genome duplication should not just be neglected.

**Results:**

In this study, we obtained EP300 protein sequences of representative teleosts, mammals and sauropsids, with which we inferred a highly supported maximum likelihood tree. We observed that Ep300 duplicates (Ep300a and Ep300b) were widely retained in teleosts and universally expressed in a variety of tissues. Consensus sequences of Ep300a and Ep300b had exactly the same distribution of conserved domains, suggesting that their functions should still be largely overlapped. We analyzed the molecular evolution of Ep300 duplicates in teleosts, using branch-site models, clade models and site models. The results showed that both duplicates were subject to strong positive selection; however, for an extant species, generally at most one copy was under positive selection. At the clade level, there were evident positive correlations between evolutionary rates, the number of positively selected sites and gene expression levels. In Ostariophysi, Ep300a were under stronger positive selection than Ep300b; in Neoteleostei, another species-rich teleost clade, the contrary was the case. We also modeled 3D structures of zf-TAZ domain and its flanking regions of Ep300a and Ep300b of *D. rerio* and *Oryzias latipes* and found that in either species the faster evolving copy had more short helixes.

**Conclusions:**

Collectively, the two copies of Ep300 have undoubtedly experienced directional divergence in main teleost clades. The divergence of EP300 between teleosts and mammals should be greater than the divergence between different teleost clades. Further studies are needed to clarify to what extent the EP300 involved regulatory network has diverged between teleosts and mammals, which would also help explain the huge success of teleosts.

## Background

Cells under changing external environments need to regulate their transcriptions to maintain internal homeostasis, during which lysine acetylation plays a key role in connecting external signals and downstream regulations [1]. Tens of human proteins have been convincingly demonstrated to be lysine acetyltransferases (KATs), which, together with their complexes, are recruited in a context-specific and cell type-specific manner to particular genomic elements (promoters, enhancers and gene bodies) to modulate the transcriptional output required for proper development and housekeeping functions. Apart from histones in chromatin, thousands of proteins are also substrates of KATs, which are distributed in the cell nucleus, cytoplasm, mitochondria, endoplasmic reticulum and peroxisomes. Acetylation of these proteins can change their cellular localization, enzymatic activity and their ability to interact with other protein complexes [1, 2]. Of the many KATs, the EP300/CREBBP family has been reported to play an essential role in the HIF-1α pathway that responds to hypoxia stress [3]. Since hypoxia is a common character in many types of solid tumors [4], EP300/CREBBP and HIF-1α that confer a survival pathway for hypoxic tumor cells have been heavily studied as cancer drug targets [5, 6]. EP300 and CREBBP originated from a whole genome duplication (WGD) event that occurred in the common ancestor of vertebrates more than 450 million years ago (MYA) [5, 7]. Having diverged for such a long time, their structures are still highly similar: they display 57% identity at the whole protein level and 88% similarity in their core KAT domains [8]. Besides the KAT domain, they also share other non-catalytic conserved regions: a nuclear receptor interacting domain (NRID) at the N-terminal side; three cysteine-histidine (CH)-rich domains (CH1, CH2 and CH3), of which the CH1 and CH3 contain transcriptional adaptor zinc fingers (TAZ1 and TAZ2) and the CH3 also contains a ZZ zinc finger, while the CH2 is part of the catalytic KAT domain and contains a plant homeodomain (PHD) and an interleaved RING domain; a KIX domain and a bromodomain between the CH1 and CH2; a nuclear receptor coactivator binding domain (NCBD) at the C-terminal side [6, 8, 9]. Based on these domains, both EP300 and CREBBP contain at least nine protein-binding sites for a huge variety of proteins, including TFs, kinases, chromatin remodelers, structural proteins and others [10]. The structural similarity between EP300 and CREBBP makes their functions largely overlapping. Yet there is increasing evidence that they also serve unique functions, which may be due to slight differences in substrate specificity or a subset of protein-protein binding interactions or both [11].

*Danio rerio* (zebrafish) is a useful model organism to study genetic diseases and test new drugs due to its transparent embryos and fast growing speed [12–14]. However, we should be cautious in conducting experiments and interpreting results when EP300 or CREBBP is involved. In addition to two rounds of WGD events occurred in the common ancestor of vertebrates, a third round of WGD event occurred in the common ancestor of teleosts about 320–350 MYA [15–17]. It was estimated that approximately 80% of the duplicated genes lost one copy in a very short time [18]. However, by searching against the TreeFam database, we found that the two copies of both EP300 and CREBBP are likely to be widely retained in teleosts [19, 20]. As “master coactivators” in regulatory networks [1, 2], the initial reason why duplicates of EP300 and CREBBP are retained is most probably to maintain dosage balance [21]. It was reported that genes kept in double after genome duplication represent the subset under strongest purifying selection [22]. On the other hand, two duplicates generally evolve asymmetrically [22], which will finally lead to functional divergence and even gene separation (genesis of new genes). By searching against the Selectome database, we found that both EP300 and CREBBP of teleosts have positively selected branches, while mammals and sauropsids have none [23–26]. If EP300 and CREBBP of teleosts have experienced strong and constant positive selection, they may have diverged considerably in functions from their mammalian orthologs. Given the fundamental roles of EP300 and CREBBP in regulatory networks, positive selection on them may also correlate with the huge success of teleosts [27]. However, the Selectom database cannot provide more details since it only includes a limited number of teleost species and the method used is restricted to branch-site models.

In this study, we focused on the molecular evolution of Ep300 in teleosts. We are particularly interested in the way and the extent of divergence of the retained duplicates in different teleost clades, which were explored through analyses of molecular evolution of Ep300 duplicates based on diverse evolutionary models, tissue expression profiles and protein structures.

## Results

### Retention of Ep300 duplicates in teleosts

Through blastp search against NCBI nr database, we obtained 114 EP300 protein sequences from 28 fishes, 30 mammals and 25 sauropsids (a detailed list of these species and their respective lineage information can be found in Additional file 1). All mammals and sauropsids had only one copy; the fishes had 2.1 copies on average, with 21 fishes had exactly 2 copies and only one teleost fish had one copy (Additional file 2). *Sinocyclocheilus anshuiensis*, *Carassius auratus* (goldfish), *Austrofundulus limnaeus* and *Oncorhynchus kisutch* (coho salmon) had 3 ~ 4 copies, which was in accordance with the fact that their respective ancestors underwent recent genome duplications [28–30]. The two non-teleost fishes, *Erpetoichthys calabaricus* (reedfish) and *Lepisosteus oculatus* (spotted gar), had only one copy. We also conducted tblastn search against RefSeq genomes of 19 fish species that have available assembled chromosomes and found no evidence of any species to have additional copies that might originate from small-scale duplications (SSDs) (Additional file 3). Therefore, the best explanation is that the commonly appeared two copies in teleost fishes originated from the teleost-specific WGD. To get more direct evidence, we inferred phylogenetic trees by both maximum likelihood (ML) and Bayesian methods, both of which showed clear separation of two big teleost clades (Additional files 4 and 5). The topologies of the two trees were very similar: at least 98% of edges of one tree could be found on the other and the normalized Robinson-Foulds distance was 0.03 (see Table S1 in Additional file 6). However, the Bayesian tree unreasonably placed Ep300a of *Scleropages formosus* (Asian bonytongue) and *Paramormyrops kingsleyae* (Additional file 5); therefore, only the ML tree was used for further analyses.

**Fig. 1:**
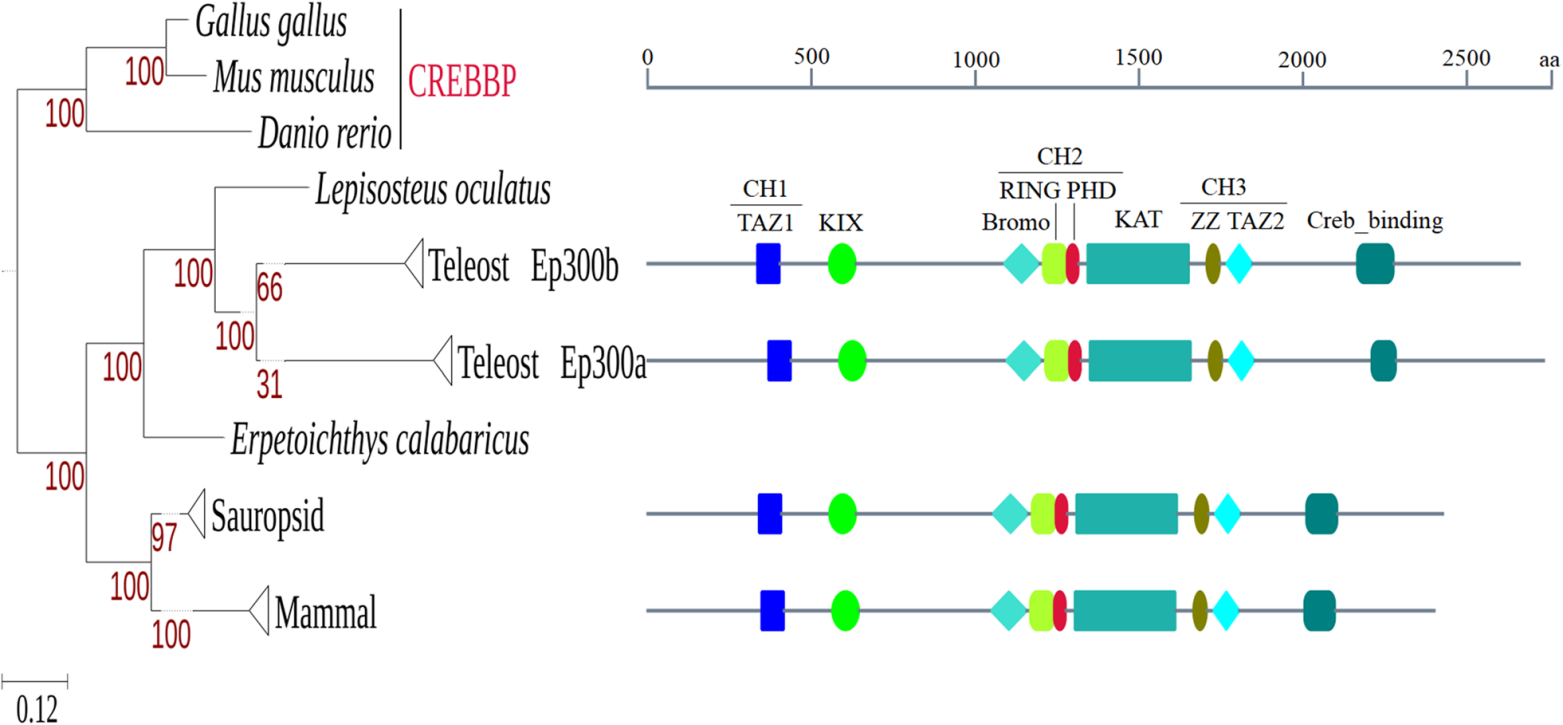
A collapsed ML tree of EP300 and conserved domains within consensus sequences. The original tree can be seen in Additional file 4. From left to right, the displayed conserved domains were zf-TAZ (pfam02135), KIX (pfam02172), Bromo_cbp_like (cd05495), RING_CBP-p300 (cd15802), PHD_p300 (cd15646), HAT_KAT11 (pfam08214), ZZ_CBP (cd02337), ZnF_TAZ (smart00551) and Creb_binding (pfam09030). The Creb_binding domain annotated by CDD search was called nuclear receptor coactivator binding domain (NCBD) or interferon binding domain (IBiD) elsewhere [6, 8]. CH1-3 indicate three cysteine-histidine-rich domains

We also generated consensus protein sequences of Ep300a and Ep300b of teleost fishes and EP300 of mammals and sauropsids. We queried the conserved domains within these consensus sequences and found that Ep300a and Ep300a had exactly the same distribution of conserved domains as EP300 of mammals and sauropsids (Fig. 1). That is not unexpected since EP300 and CREBBP are also highly similar to each other (Additional file 6, Fig. S1 and S2) [31]. It should be noted, however, that conserved domains shown in Fig. 1 were just specific hits reported by CDD search; there were also non-specific hits called superfamilies (Additional file 6, Fig. S3–S8). For specific species, two copies of Ep300 can differ in both specific hits and superfamilies. Take *D. rerio* as an example: its Ep300b did not have the specific PHD_p300 domain that existed in Ep300a, but had a PHD_SF superfamily in the corresponding region (Additional file 6, Fig. S5 and S6).

### Ep300a and Ep300b were widely subject to positive selection

**Fig. 2:**
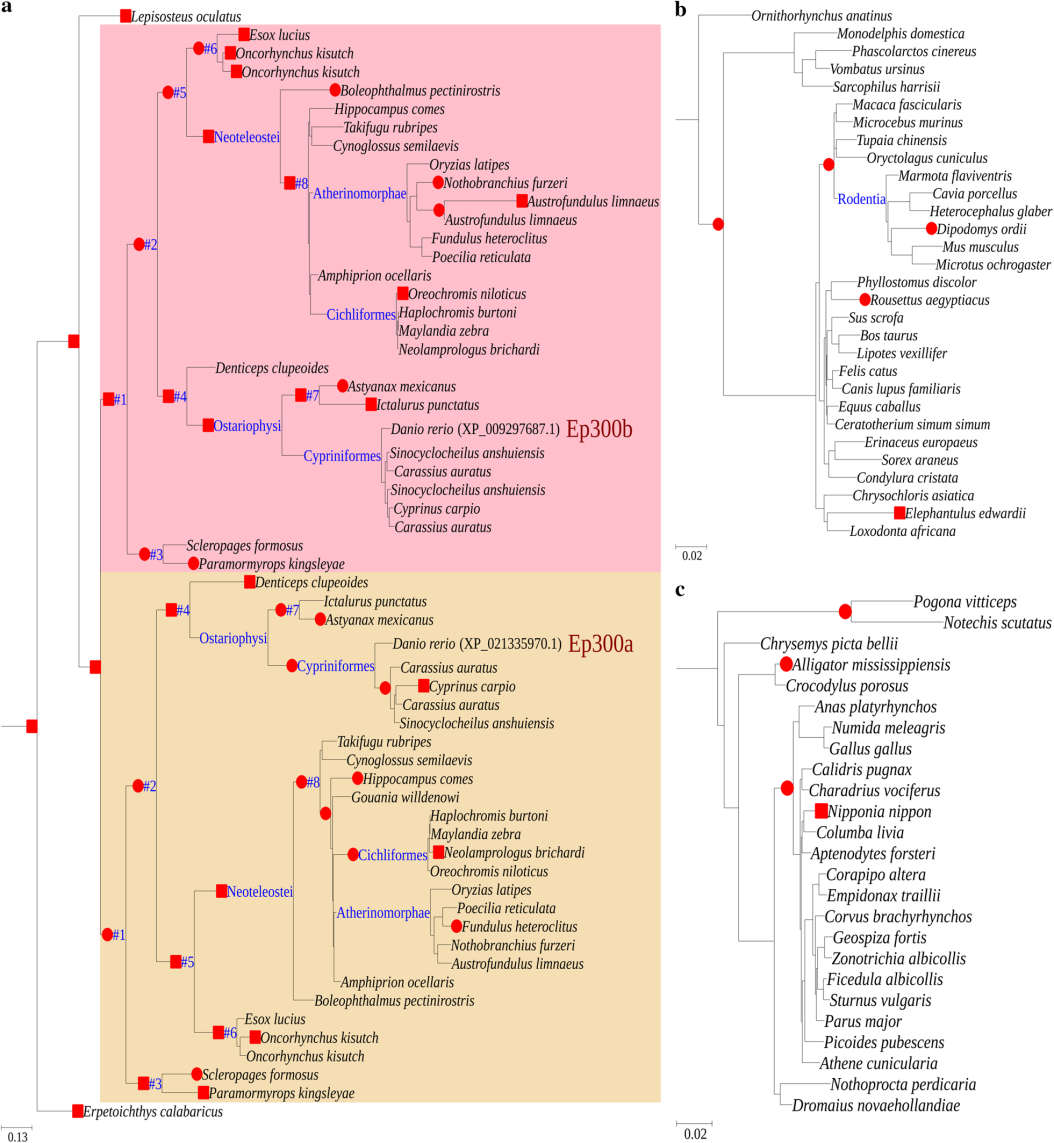
Positively selected branches reported by branch-site test. Fishes (**a**), mammals (**b**) and sauropsids (**c**) are displayed separately. A red square indicates that the multiple testing corrected *p* value was lower than 0.05; a red circle indicates that the multiple testing corrected *p* value was higher than 0.05, but the uncorrected *p* value was lower than 0.05

We used aBSREL [32] to test branches that were subject to episodic positive selection throughout the ML tree (Fig. 2). Since multiple testing greatly reduces the statistical power in an exploratory analysis, we considered all branches with uncorrected *p* value lower than 0.05. Of the 55 branches of Ep300a, 22 were under positive selection; of the 57 branches of Ep300b, 21 were under positive selection. By contrast, the proportions of positively selected branches of mammals and sauropsids were 5/59 and 4/49, respectively. The proportion of positively selected branches of either Ep300a or Ep300b was very significantly higher than that in mammals and sauropsids (*p* values all lower than 0.01, see Table S2 in Additional file 6); however, the difference between the two copies was not significant (all comparisons were conducted by Fisher’s exact test [33]). Of the 43 positively selected branches of Ep300a and Ep300b, over half were internal branches, viz. common ancestors. Furthermore, in 9 ancestral species (branches #1–#8 and Neoteleostei, which were all dated back to more than 100 MYA), both duplicates were under positive selection, while in most extant species, only one or none duplicate was under positive selection.

When we inspected the *ω* ratio of every branch (reported by aBSREL test; see Additional file 7), we observed that the type of asymmetric evolutionary rates of Ep300a and Ep300b was different in different species: e.g. in *D. rerio* Ep300a evolved faster than Ep300b, while in *Oryzias latipes* the contrary was the case. From the common ancestor #1 to the majority of extant species, there was not a constant trend of which copy evolves faster.

### Faster evolving Ep300a/Ep300b contained more positively selected sites

We used Clade model C (CmC) [34] and RELAX [35] to compare overall evolutionary rates of Ep300a and Ep300b in different clades. One significant difference between CmC and RELAX is that the latter incorporates rate variation in synonymous sites (*d*s) across sites and branches. Still, their results were accordant: in each comparison, a higher *ω*_2_ in CmC result was accompanied by a higher mean *ω* value in RELAX result (Table 1). Both duplicates evolved faster than mammals and sauropsids, which was in accordance with previous results [22, 36]. At teleost level, the two duplicates evolved at almost the same rate; *p* values of CmC test and RELAX test were not sufficiently small either (Table 1). In four smaller clades (Neoteleostei, Atherinomorphae, Ostariophysi and Cypriniformes), the two duplicates evolved at significantly different rates and with very low *p* values: in the clade Neoteleostei and its subclade Atherinomorphae, Ep300b evolved faster than Ep300a; in the clade Ostariophysi and its subclade Cypriniformes, however, Ep300a evolved faster than Ep300b. Although the duplicates generally evolved at different rates, the moderate k values reported by RELAX indicated that there was no strong evidence of one copy to be under intensified or relaxed selection relative to the other.

**Table 1:** Results of CmC and RELAX analyses

Clade	CmC					RELAX			
	branch label^a^	*ω*_0_	*ω*_1_	*ω*_2_	*p*^b^	branch label	mean *ω*	k^c^	*p*
Mammal	#1	0.01304 (70.9%)	1 (1.1%)	0.24285 (28.0%)		Test	0.0794		
Sauropsid	#2	0.01304 (70.9%)	1 (1.1%)	0.16454 (28.0%)	5.72e-14	Reference	0.0553	0.78	3.31e-14
Teleost (Ep300a)	#1	0.02232 (56.6%)	1 (2.9%)	0.22425 (40.5%)		Test	0.1122		
Teleost (Ep300b)	#2	0.02232 (56.6%)	1 (2.9%)	0.23696 (40.5%)	0.04754	Reference	0.1230	1.08	0.00124
Neoteleostei (Ep300a)	#1	0.02234 (56.4%)	1 (3.0%)	0.16996 (40.5%)		Test	0.0899		
Neoteleostei (Ep300b)	#2	0.02234 (56.4%)	1 (3.0%)	0.24039 (40.5%)	1.64e-33	Reference	0.1280	1.14	1.25e-12
Atherinomorphae (Ep300a)	#1	0.02225 (56.5%)	1 (2.9%)	0.16380 (40.6%)		Test	0.0907		
Atherinomorphae (Ep300b)	#2	0.02225 (56.5%)	1 (2.9%)	0.26161 (40.6%)	1.28e-14	Reference	0.1569	1.06	0.31731
Cichliformes (Ep300a)	#1	0.02222 (56.5%)	1 (2.9%)	0.26189 (40.6%)		Test	0.1513		
Cichliformes (Ep300b)	#2	0.02222 (56.5%)	1 (2.9%)	0.24542 (40.6%)	0.13992	Reference	0.1335	1.20	0.01602
Ostariophysi (Ep300a)	#1	0.02208 (56.4%)	1 (3.0%)	0.31933 (40.6%)		Test	0.1553		
Ostariophysi (Ep300b)	#2	0.02208 (56.4%)	1 (3.0%)	0.25481 (40.6%)	6.52e-20	Reference	0.1228	0.95	5.10e-06
Cypriniformes (Ep300a)	#1	0.02173 (56.1%)	1 (3.0%)	0.41002 (40.9%)		Test	0.2036		
Cypriniformes (Ep300b)	#2	0.02173 (56.1%)	1 (3.0%)	0.24213 (40.9%)	1.72e-27	Reference	0.1204	4.41	2.25e-11

^a^The labels “#1” and “#2” here have nothing to do with the same labels in Fig. 2

^b^The *p* value here is an indication of whether the CmC model is significantly better than the M2a_rel model

^c^A k > 1 combined with a *p* < 0.05 indicates that the selection strength has been intensified in the test branches relative to the reference; a k < 1 combined with a *p* < 0.05 indicates that the selection strength has been relaxed in the test branches relative to the reference

To get a thorough exploration of positively selected sites of Ep300a and Ep300b, we used MEME [37] to detect sites subject to episodic positive selection and FUBAR [38] and M8&M7 models [39] to detect sites subject to pervasive positive selection. To speculate the possible consequences of positively selected sites, we matched their positions with conserved domains of the consensus sequence (of the full sequence set). As shown in Table 2, mammals and sauropsids had much fewer positively selected sites than teleosts, which was consistent with the above results that they also had fewer positively selected branches and slower evolutionary rates. At any clade, we can find that the dominant proportion of positively selected sites was detected by MEME, confirming that natural selection is predominantly episodic [37]. An unexpected observation was that there were much fewer detected positively selected sites in big clades like teleosts than in smaller clades like Neoteleostei or Ostariophysi, suggesting that more data will not necessarily provide greater power to detect positive selection [37]. In either big or small clades of teleosts, positively selected sites detected in two duplicates were generally non-redundant, indicating that they were subject to divergent selection. In smaller clades, it was evident that the copy with a faster evolutionary rate generally had more positively selected sites. However, these positively selected sites were most commonly appeared in non-conserved regions, especially in regions before zf-TAZ domain and between KIX and Bromo_cbp_like domains.

**Table 2:** Distribution of detected positively selected sites

Clade	Positively selected sites
Mammal	(), **()zf-TAZ**, (549^E^), **()KIX**, (668^E^, 817^E^, 898^F^, 1015^M^, 1078^M^), **()Bromo_cbp_like**, (1221^E^), **()RING_CBP-p300**, **(1323**^**E**^**)PHD_p300**, (1346^E^), **(1369**^**E**^, **1510**^**E**^**)HAT_KAT11**, (), **()ZZ_CBP**, (), **()ZnF_TAZ**, (), **(2174**^**E**^**)Creb_binding**, (2280^E^, 2328^E^)
Rodentia	(267^FM^, 318^E^), **()zf-TAZ**, (486^E^, 545^E^, 549^E^, 550^E^), **()KIX**, (851^E^, 875^E^, 1015^M^, 1053^M^), **()Bromo_cbp_like**, (), **()RING_CBP-p300**, **()PHD_p300**, (1342^E^, 1344^E^, 1346^E^), **()HAT_KAT11**, (), **()ZZ_CBP**, (), **()ZnF_TAZ**, (), **(2086**^**E**^**)Creb_binding**, ()
Sauropsid	(), **()zf-TAZ**, (), **()KIX**, (817^E^, 1044^M^, 1079^E^), **()Bromo_cbp_like**, (), **()RING_CBP-p300**, **()PHD_p300**, (), **()HAT_KAT11**, (1704^E^), **(1740**^**E**^**)ZZ_CBP**, (), **()ZnF_TAZ**, (2083^E^), **()Creb_binding**, (2187^E^, 2189^E^)
Teleost (Ep300a)	(11^E^, 12^E^, 15^E^, 66^E^, 155^E^, 173^E^, 236^E^, 256^E^, 268^E^, 315^E^), **()zf-TAZ**, (500^E^), **()KIX**, (797^E^, 925^E^, 1089^M^), **()Bromo_cbp_like**, (), **(1293**^**E**^**)RING_CBP-p300**, **()PHD_p300**, (), **(1463**^**E**^**)HAT_KAT11**, (), **()ZZ_CBP**, (), **()ZnF_TAZ**, (1981^E^, 2031^E^), **()Creb_binding**, ()
Teleost (Ep300b)	(2^E^, 4^E^, 12^E^, 42^E^, 125^E^, 267^E^, 275^E^, 330^E^), **(397**^**E**^**)zf-TAZ**, (485^E^, 498^E^, 505^E^), **()KIX**, (735^E^, 832^M^, 880^E^, 910^M^, 911^M^), **(1135**^**E**^**)Bromo_cbp_like**, (), **()RING_CBP-p300**, **(1329**^**E**^**)PHD_p300**, (), **()HAT_KAT11**, (), **()ZZ_CBP**, (), **()ZnF_TAZ**, (), **(2109**^**E**^, **2152**^**E**^**)Creb_binding**, (2327^E^)
Neoteleostei (Ep300a)	(66^E^, 155^E^, 165^E^), **()zf-TAZ**, (500^E^, 575^E^), **(581**^**E**^**)KIX**, (715^E^, 765^E^, 768^E^, 812^E^, 850^E^, 1011^E^), **()Bromo_cbp_like**, (), **()RING_CBP-p300**, **()PHD_p300**, (), **(1463**^**E**^**)HAT_KAT11**, (), **()ZZ_CBP**, (), **()ZnF_TAZ**, (1968^E^), **()Creb_binding**, (2188^E^, 2242^E^)
Neoteleostei (Ep300b)	(2^E^, 4^E^, 28^E^, 37^E^, 42^E^, 271^E^, 303^E^, 313^E^), **(360**^**E**^, **397**^**E**^**)zf-TAZ**, (485^E^, 487^E^, 498^E^, 535^E^), **()KIX**, (721^E^, 759^E^, 926^E^, 932^E^, 1009^E^, 1085^E^), **()Bromo_cbp_like**, (), **()RING_CBP-p300**, **(1329**^**E**^**)PHD_p300**, (), **()HAT_KAT11**, (), **()ZZ_CBP**, (), **()ZnF_TAZ**, (), **(2109**^**E**^, **2152**^**E**^**)Creb_binding**, (2342^E^, 2444^E^, 2502^E^)
Atherinomorphae (Ep300a)	(51^EF^, 69^E^), **()zf-TAZ**, (537^E^), **(581**^**E**^**)KIX**, (715^E^, 719^E^, 750^E^, 887^M^, 1089^M^), **()Bromo_cbp_like**, (), **()RING_CBP-p300**, **(1303**^**F**^**)PHD_p300**, (), **()HAT_KAT11**, (), **()ZZ_CBP**, (), **()ZnF_TAZ**, (1971^E^), **()Creb_binding**, (2480^E^)
Atherinomorphae (Ep300b)	(2^E^, 12^E^, 37^E^, 115^E^, 123^M^, 127^M^, 136^E^, 138^E^, 186^E^, 197^E^, 233^E^, 269^E^, 276^E^, 280^E^, 292^M^, 303^E^, 305^E^, 336^E^, 337^E^), **(389**^**E**^**)zf-TAZ**, (473^E^, 487^E^), **()KIX**, (727^E^, 735^E^, 803^E^, 868^E^, 935^E^, 959^E^, 972^E^, 992^E^, 1009^E^, 1015^E^, 1034^E^, 1097^E^), **()Bromo_cbp_like**, (), **()RING_CBP-p300**, **(1329**^**E**^**)PHD_p300**, (), **()HAT_KAT11**, (), **()ZZ_CBP**, (), **()ZnF_TAZ**, (), **(2097**^**M**^**)Creb_binding**, (2271^E^, 2323^E^)
Cichliformes (Ep300a)	(214^E^), **()zf-TAZ**, (), **(594**^**EFM**^**)KIX**, (723^M^, 763^EF^, 765^E^, 766^EM^, 778^EM^, 781^E^, 788^EF^), **()Bromo_cbp_like**, (), **()RING_CBP-p300**, **()PHD_p300**, (), **()HAT_KAT11**, (), **()ZZ_CBP**, (), **()ZnF_TAZ**, (), **()Creb_binding**, ()
Cichliformes (Ep300b)	(), **()zf-TAZ**, (), **()KIX**, (904^M^, 910^M^, 911^F^), **()Bromo_cbp_like**, (), **()RING_CBP-p300**, **()PHD_p300**, (), **()HAT_KAT11**, (), **()ZZ_CBP**, (), **()ZnF_TAZ**, (), **()Creb_binding**, ()
Ostariophysi (Ep300a)	(11^E^, 12^E^, 14^E^, 15^E^, 66^E^, 155^E^, 259^E^, 262^E^, 266^E^, 277^E^, 282^E^, 283^E^, 331^E^), **()zf-TAZ**, (576^E^), **()KIX**, (676^M^, 727^E^, 753^E^, 762^E^, 802^E^, 805^FM^, 841^M^, 842^E^, 849^E^, 861^E^, 867^M^, 880^E^, 884^E^, 929^E^, 932^E^, 982^E^, 1002^E^, 1007^E^, 1015^E^, 1068^E^, 1090^M^, 1094^M^), **()Bromo_cbp_like**, (1223^M^), **(1293**^**E**^**)RING_CBP-p300**, **(1323**^**E**^**)PHD_p300**, (), **(1467**^**M**^, **1529**^**E**^, **1569**^**E**^**)HAT_KAT11**, (), **()ZZ_CBP**, (1773^E^), **(1810**^**E**^, **1812**^**E**^**)ZnF_TAZ**, (1892^E^, 1900^E^, 2038^E^, 2059^E^), **(2098**^**E**^, **2121**^**FM**^, **2127**^**E**^, **2129**^**E**^**)Creb_binding**, (2331^E^, 2409^E^, 2423^E^, 2446^E^)
Ostariophysi (Ep300b)	(125^FM^, 179^E^, 267^E^), **(400**^**E**^**)zf-TAZ**, (), **()KIX**, (708^E^, 735^E^, 740^E^, 822^M^, 1099^E^), **(1135**^**E**^**)Bromo_cbp_like**, (), **()RING_CBP-p300**, **()PHD_p300**, (1339^E^), **()HAT_KAT11**, (1671^E^), **(1754**^**E**^**)ZZ_CBP**, (), **()ZnF_TAZ**, (1982^E^), **(2106**^**E**^, **2119**^**E**^**)Creb_binding**, (2498^E^)
Cypriniformes (Ep300a)	(9^E^, 12^E^, 15^E^, 66^E^, 155^M^, 262^E^, 266^E^, 268^E^, 277^E^, 282^E^), **()zf-TAZ**, (), **()KIX**, (676^FM^, 689^E^, 762^E^, 802^E^, 805^M^, 841^E^, 849^EM^, 852^E^, 880^E^, 885^E^, 887^FM^, 929^E^, 936^FM^, 1002^E^, 1007^EF^, 1064^E^, 1068^EF^, 1090^M^), **()Bromo_cbp_like**, (), **()RING_CBP-p300**, **()PHD_p300**, (), **(1463**^**M**^**)HAT_KAT11**, (), **()ZZ_CBP**, (1773^E^), **(1810**^**E**^, **1812**^**EFM**^, **1813**^**E**^**)ZnF_TAZ**, (), **(2098**^**E**^**)Creb_binding**, (2196^EF^, 2409^E^, 2423^E^)
Cypriniformes (Ep300b)	(125^FM^, 267^E^), **()zf-TAZ**, (), **()KIX**, (740^E^, 822^FM^), **()Bromo_cbp_like**, (), **()RING_CBP-p300**, **()PHD_p300**, (), **()HAT_KAT11**, (1671^EF^), **(1754**^**FM**^**)ZZ_CBP**, (), **()ZnF_TAZ**, (), **()Creb_binding**, (2215^E^, 2470^E^)

A pair of parentheses without a following name indicates that they contain positively selected sites outside conserved domains; whereas a pair of parentheses with a following name (like zf-TAZ) indicates that they contain positively selected sites located inside a conserved domain. The numbers in parentheses indicate the position of positively selected sites; the superscripts (E, F and M) of a number indicate which method reported this position, with E to represent MEME, F to represent FUBAR and M to represent M8&M7 models

### Structural features of zf-TAZ domain and its flanking regions

**Fig. 3:**
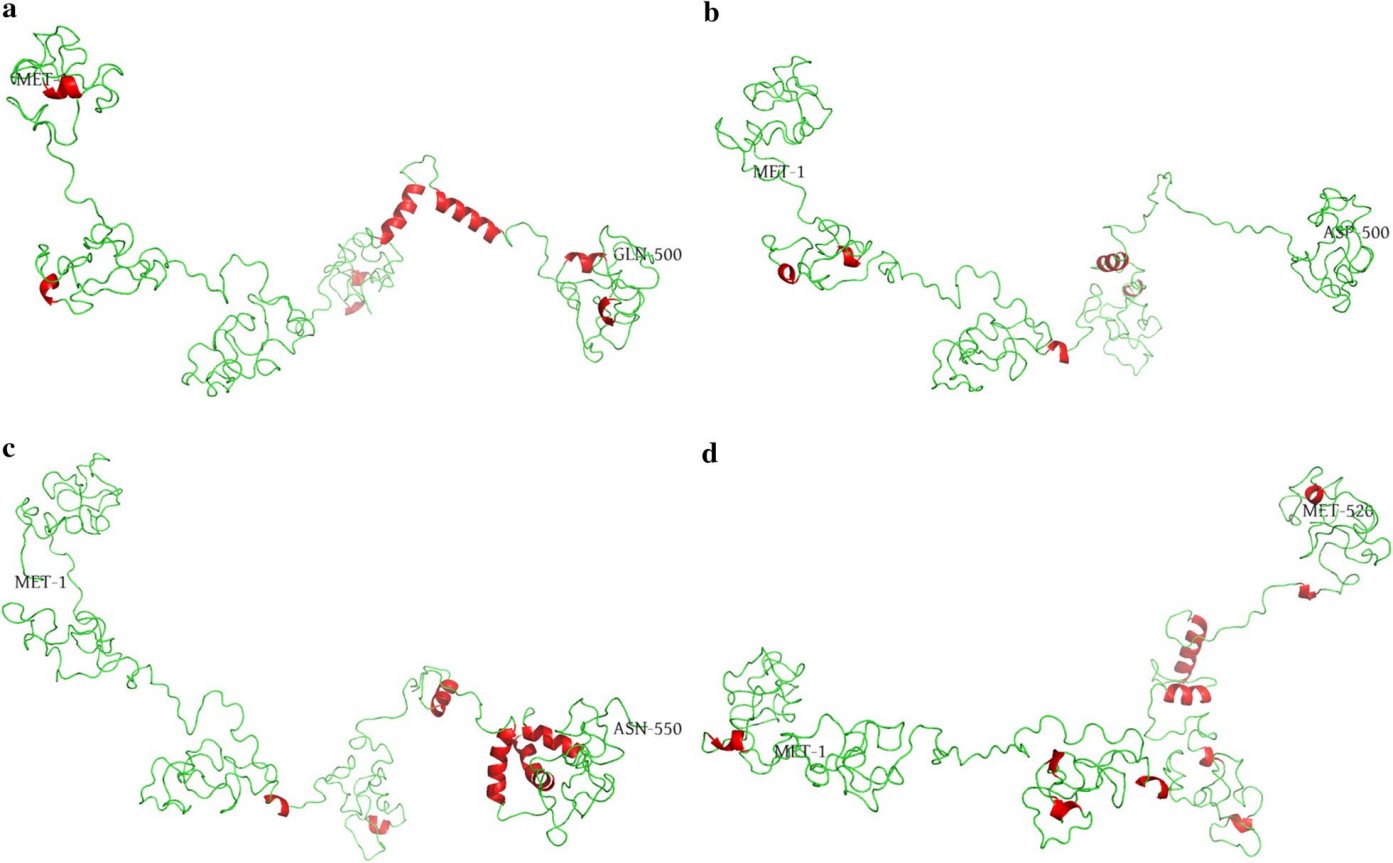
Structures of the zf-TAZ domain and its flanking regions. The source sequences were Ep300a of *D. rerio* (**a**), Ep300b of *D. rerio* (**b**), Ep300a of *O. latipes* (**c**) and Ep300b of *O. latipes* (**d**). The first (always MET-1) and last residue of each sequence used for modeling are labeled; α-helixes are colored red

We modeled structures of zf-TAZ domain and its flanking regions of Ep300a and Ep300b of *D. rerio* and *O. latipes* (as representatives of Ostariophysi and Neoteleostei, respectively) using I-TASSER suit. All four best models of respective sequences had significantly greater structure density (by the number of decoys) than respecti,ve lower-rank models; three of them even had TM-score greater than 0.5 (see Table S3 in Additional file 6). In three best models (except for Ep300b of *D. rerio*), the region corresponding to zf-TAZ domain was characterized by long α-helixes, further confirming the credibility of the best models (Fig. 3). In flanking regions, especially the N-terminal side, short helixes were frequently appeared. Ep300a of *D. rerio* and Ep300b of *O. latipes*, which evolved faster and had more positively selected sites, also contained more short helixes than their respective paralogs (Fig. 3). It should be noted that the flanking regions are mainly loops, the modeling of which would heavily rely on remote homology (or ab initio modeling) and consequently may not be very stable. We also modeled structures of the N-terminal side flanking region of zf-TAZ domain and found that the number and distribution of short helixes changed greatly in best models of this region (Additional file 6, Table S4 and Fig. S9). However, Ep300a of *D. rerio* and Ep300b of *O. latipes* still contained more short helixes than their respective paralogs.

### Correlation between tissue expression profile and evolutionary rate

**Fig. 4:**
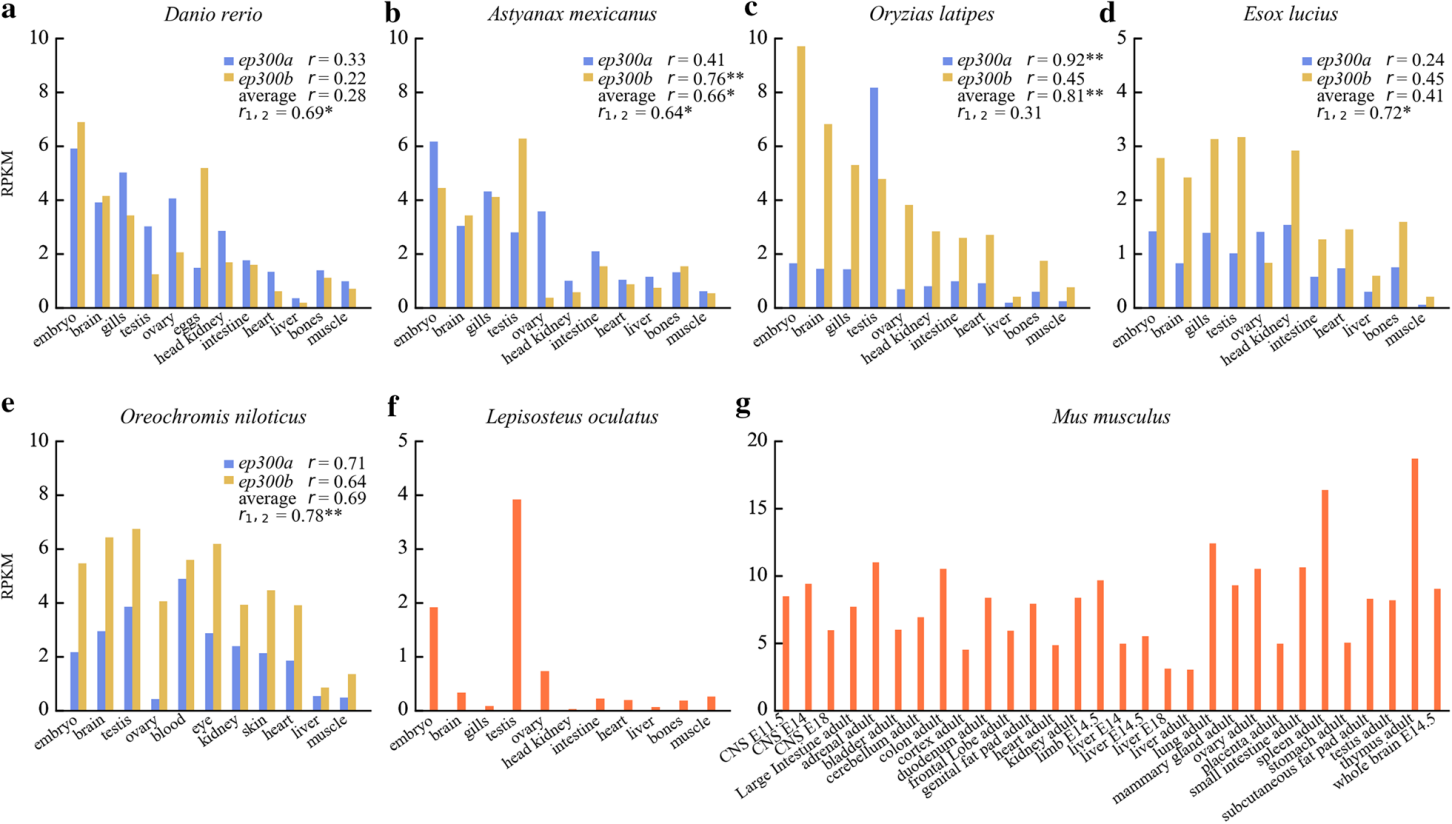
Tissue expression profiles of *ep300* genes of different species. Four types of Pearson correlation coefficient (*r*) values were calculated: an *r* value following “*ep300a*” indicates the correlation between *ep300a* of a teleost and *ep300* of *L. oculatus*; an *r* value following “*ep300b*” indicates the correlation between *ep300b* of a teleost and *ep300* of *L. oculatus*; an *r* value following “average” indicates the correlation between average values of *ep300a* and *ep300b* of a teleost and *ep300* of *L. oculatus*; *r*_1,2_ indicates the correlation between *ep300a* and *ep300b* of a teleost. A single asterisk (*) indicates that the correlation was significant (*p* < 0.05); two asterisks (**) indicate that the correlation was very significant (*p* < 0.01)

We analyzed the tissue expression profiles of *ep300a/ep300b* of five teleosts, *D. rerio*, *Astyanax mexicanus* (Mexican tetra), *O. latipes*, *Esox lucius* (northern pike) and *Oreochromis niloticus* (Nile tilapia); *ep300* of one holostean fish, *L. oculatus*, which had not been affected by the teleost-specific WGD event; and *Ep300* of *Mus musculus* (house mouse) as control. In three teleosts (*D. rerio*, *E. lucius* and *O. niloticus*), tissue expression profiles of *ep300a/ep300b* did not correlate with *ep300* of *L. oculatus*, neither individually nor on average (Fig. 4). The extraordinarily high level of *ep300b* of *A. mexicanus* and *ep300a* of *O. latipes* in testis made their tissue expression profiles significantly correlate with *ep300* of *L. oculatus*. Simply removing the testis expression data will make the correlations not significant. On the other hand, in all five teleosts, the expression profiles of the two copies were significantly correlated (for *O. latipes*, the testis expression data should be excluded), suggesting that their functions have not sufficiently diverged yet. Compared to teleosts and *M. musculus*, *ep300* gene of *L. oculatus* was highly expressed in a smaller subset of tissues. According to the PhyloFish database, the quality of sequencing data of *L. oculatus* was not significantly inferior to that of other fishes (see Table S5 in Additional file 6).

We also found that tissue expression profiles of the two duplicates correlated with evolutionary rates: in four fishes (*D. rerio*, *A. mexicanus*, *O. latipes* and *E. lucius*) where one copy evolved faster than the other (Additional file 7), the copy with a faster rate had higher gene expression levels in more tissues (Table 1 and Fig. 4). At Neoteleostei level, Ep300b evolved faster than Ep300a; therefore even for *O. niloticus* of which the duplicates evolved at similar rates, the above correlation between evolutionary rate and gene expression levels still holds true. We further analyzed tissue expression profiles of *ep300a/ep300b* of *D. rerio* and *O. latipes* based on the NCBI SRA Study ERP121186. The relative abundance of *ep300a* and *ep300b* were largely in accordance with the above observations (Additional file 6, Table S6).

## Discussion

It has been widely acknowledged that the most common fate of duplicates originated from WGD is loss of one copy and becoming singleton again [22, 30, 40]. Duplicates may be successfully retained due to subfunctionalization, neofunctionalization and requirement to keep dosage balance [21, 30]. It was reported that in *D. rerio*, the Ep300b KAT domain does not have detectable acetyltransferase activity [41]. In this study, we found that Ep300b of *D. rerio* even lost the conserved PHD_p300 domain (Additional file 6, Fig. S6), which could be found in EP300 of mammals and Ep300a/Ep300b of *O. latipes*. It has been reported that PHD domain, together with other domains that flank the KAT domain, regulates acetyltransferase activity and also promotes SUMOylation of the adjacent CRD1 cell cycle regulatory domain [9]. The loss of the PHD_p300 domain is likely to be an important cause of the inactivation of KAT domain in Ep300b of *D. rerio*. Meanwhile, it was suggested that the transcriptional coactivator function of Ep300b (conferred by other conserved domains) might still play subtle but important roles in development [41]. Therefore, Ep300b of *D. rerio* had undoubtedly experienced subfunctionalization. On the other hand, we observed that the faster evolving copy of Ep300a/Ep300b generally contained more positively selected sites and more structural innovations (short helixes) in the most intensively selected regions (Tables 1, 2, Fig. 3), suggesting that they had also been subject to neofunctionalization. The moderate k values reported by RELAX means that selective constraints acted on the duplicates were largely the same (Table 1); therefore, divergence between the duplicates is not likely to cause significant subfunctionalization or significant neofunctionalization in short periods of time, but fine tuning of them both. Still, it seems that teleosts favor functional innovations: in five representative species, the faster evolving copy had higher expression levels in more tissues (Table 1 and Fig. 4). From tissue expression profiles we can also conclude that Ep300 of *L. oculatus* is still very primitive, while its orthologs in mammals and teleosts are more finely tuned (Fig. 4). Since the divergence between teleosts and *L. oculatus* occurred later than the divergence between teleosts and mammals [16], the paths teleosts and mammals took to tune functions of EP300 may be distinct, which will inevitably affect the tuning results.

Constituting around half of all vertebrate species, teleosts are by far the most successful vertebrate clade [27]. Given the fact that teleosts and some other diverse taxa have all experienced WGD events before their radiation [42, 43], it was thought that there is a causal correlation between WGD, evolutionary success and radiation [30]. However, the universal time delay between WGD and phases of radiation [44–46] suggests that WGD itself has not been the direct factor generating diversity. It is more likely that duplication and subsequent divergence of some essential genes enabled by WGD directly facilitate radiation [30]. Cells of multicellular organisms generally contain the same DNA sequence, yet they are differentiated into extremely diverse cell types that differ in both structure and function. This variation is largely due to the transcriptional activity of different sets of genes in different cell types, which is closely controlled by a number of epigenetic mechanisms, including acetylation, methylation, non-coding RNAs, etc. [6, 47]. Thousands of proteins have recently been identified as substrates of EP300, indicating that their binding affinities are generally weak [2, 6]. Even slight changes in EP300 structure might have profound effects on substrate specificity. Therefore, the expanded number and diversified function of Ep300 in teleosts should enable more sophisticated transcriptional regulation and additional morphological diversity, and finally facilitate their radiation. The question is how tightly they are correlated. In this study, we observed that the evolutionary process of Ep300a/Ep300b had coincided with the radiation of teleosts. In early stages, there were enough ecological niches to occupy; therefore natural selection should favor evolutionary innovation of both copies to explore a wider subset of the phenotypic space. Correspondingly, we found that duplicates of many ancestral species were both subject to positive selection (Fig. 2). As the number of species increases, ecological niches are tending to be exhaustively partitioned, which would decrease the requirement for innovation. Consequently, we found that in most extant species at most one copy was under positive selection (Fig. 2). In Ostariophysi and Neoteleostei, the two most species-rich teleost clades [30, 45], the directions of divergence of Ep300 duplicates were just the opposite, suggesting that their requirements for fine tuning of Ep300 controlled transcriptional regulation were also divergent. A deeper reason for this directional divergence is possibly the ecological segregation of their respective ancestors. For either Ep300a or Ep300b, natural selection mainly acted on regions flanking conserved domains (Table 2); however, these regions are not just linkers that allow flexible spatial arrangement of the structured domains, they can also bind transcription factors [48]. Therefore, positively selected sites detected in Ep300 duplicates can more or less change their binding affinity to substrates, alter transcriptional regulation, and finally facilitate radiation of teleosts.

## Conclusions

The importance of EP300 as a key mediator of cellular homeostasis has been well established, yet the knowledge about the divergence of EP300 between teleosts and mammals is very limited, which will inevitably affect the effectiveness of using *D. rerio* as a model organism to study EP300 related diseases and drugs. In this study, we found that WGD derived duplicates of Ep300 were widely retained in teleosts. In representative teleosts, the two copies were both expressed in many tissues, suggesting that their functions were also widely retained. Based on analyses of positively selected branches, positively selected sites, relative evolutionary rates, protein structures and tissue expression profiles, we observed divergent evolution of Ep300 duplicates in teleosts. The directions of divergence of Ep300 duplicates in Ostariophysi and Neoteleostei were just the opposite, suggesting that tuning functions of Ep300 duplicates may promote adaptation to new ecological niches and speciation of teleosts. The divergence of EP300 between teleosts and mammals should be greater than between different teleost clades. Further studies are needed to clarify the difference of EP300 involved regulatory network between teleosts and mammals.

## Methods

### Obtainment of EP300 homologs

To obtain homologs of EP300 from interested species (detailed information is listed in Additional file 1), we selected *D. rerio*, *M. musculus* and *Gallus gallus* as representatives of bony fishes (NCBI:txid7898), mammals (NCBI:txid40674) and sauropsids (NCBI:txid8457), respectively. The protein sequences (Genbank accession No.: XP_021335970.1, NP_808489.4 and XP_004937767.1) of the above three species were used as query sequences to conduct blastp search against nr database of their respective clade, with the max target sequences set to be 20,000 and expect threshold to be 1e-5.

To extract sequences of interested species from the blastp results, we first filtered non-wanted hits: if the word “p300” did not appear in the description of hit sequence or if the source organism was not interested, this hit would be ignored. Then we extracted the NCBI gene id, sequence status (validated, model, etc.) and respective nucleotide sequence accession number of each hit from the file “gene2accession” (downloaded from ftp://ftp.ncbi.nlm.nih.gov/gene/DATA/). The remaining hits would be selected based on gene ids: for each gene id, if none sequence had the status “VALIDATED”, the top hit would be selected; if at least one sequence had the status “VALIDATED”, the top hit of them would be selected.

### Phylogenetic analyses

Based on the above information (see Additional file 2), we downloaded the protein and nucleotide sequences of each selected hit/gene. We also added CREBBP sequences (of *D. rerio*, *M. musculus* and *G. gallus*) into the EP300 sequences to serve as outgroup. After that, the protein sequences were subject to multiple sequence alignment by MAFFT [49], using the L-INS-i method. The alignment was trimmed to the average length of the original sequences by removing columns with excessive gaps [50].

We used RAxML 8.2.8 [51] and MrBayes 3.2.7 [52] to infer phylogenetic trees from the trimmed alignment, respectively. The RAxML tree was inferred using the GAMMA model of rate heterogeneity, automatically determined substitution model and 500 bootstraps. The Bayesian tree was inferred under mixed models, run for 1 million generations with defaulted 25% burn-in and two parallel analyses. To use Metropolis coupling to improve the MCMC sampling of the target distribution, the Nchains parameter was set to be 12. Convergence was confirmed by checking that the standard deviations of split frequencies approached zero (below 0.01) and that there was no obvious trend in the log likelihood plot. The topologies of the two trees were compared with ete-compare [53]. The alignment files and original tree files are supplied in Additional file 8.

### Molecular evolutionary analyses

Before conducting evolutionary analyses, we arranged the nucleotide sequence alignment based on the trimmed protein sequence alignment described above. To estimate episodic positive selection acting on specific branches, we performed aBSREL test [32] with HYPHY 2.5.0 [54] on all branches within a tree. To compare selective pressures of duplicates at different clade levels, we performed CmC test [34] with codeml program from the PAML 4 software package [55] and RELAX test [35] with HYPHY. In a CmC test, all internal and external branches of two interested clades in a tree file were labeled with “#1” and “#2”, respectively; in a RELAX test, the labels were “Test” and “Reference”, respectively. To estimate sites subject to positive selection, we first extracted subtrees containing only interested species; respective nucleotide sequences were also extracted from alignments of full sequence set. We performed M8&M7 models test [39] with codeml program and FUBAR test [38] with HYPHY to estimate sites subject to pervasive positive selection. To estimate sites subject to episodic positive selection, we performed MEME test [37] with HYPHY, in which we consider all branches of a subtree.

### Consensus sequences and conserved domains

To get a consensus sequence of the full sequence set, we extracted the most frequent residue of each column (if it is a gap, then the less frequent residue would be selected) from the trimmed protein sequence alignment. For smaller sequence sets, e.g. a set only contained mammals’ sequences, the sequences would be aligned with MAFFT first. Then the alignment would be trimmed to the average length of the original sequences (after a first calculation, sequences with length shorter than 60% of the average value would be excluded, the remaining sequences were used to calculate the final average length). The conserved domains and their exact locations within consensus sequences were predicted by the NCBI online tool CDD search (https://www.ncbi.nlm.nih.gov/Structure/cdd/wrpsb.cgi) [56].

### Modeling of protein structure

We used I-TASSER suit (version 5.1) [57] to model structures of zf-TAZ domain and its flanking regions of Ep300a and Ep300b of *D. rerio* (Genbank accession No.: XP_021335970.1 and XP_009297687.1) and *O. latipes* (Genbank accession No.: XP_023805552.1 and XP_011476788.1). For Ep300a and Ep300b of *D. rerio*, the top 500 aa were used as query sequences; for Ep300a of *O. latipes*, the top 550 aa were used as query sequence; for Ep300b of *O. latipes*, the top 520 aa were used as query sequence. The best models were visualized using PyMOL [58]. We used MAFFT to conduct multiple alignment of the four query sequences, the result of which can be seen in Additional file 9.

### Gene expression data

To get gene expression data of *ep300a/ep300b* of *D. rerio*, *A. mexicanus*, *O. latipes* and *E. lucius* and *ep300* of *L. oculatus*, the nucleotide sequence of each gene was used as a query to search against PhyloFish database [15]. The best hit was selected to further explore its own length and expression data in different tissues (indicated by the number of matched reads). The total number of RNA-seq reads of respective tissues and species were also collected from PhyloFish. The above data were combined together to calculate RPKMs of each gene in respective tissues and species. The RPKMs of *ep300a/ep300b* of *O. niloticus* and *Ep300* of *M. musculus* in different tissues were obtained from the NCBI gene database with their respective gene ids as queries (see Additional file 2). Correlation of expression profiles between duplicates (and between *ep300a/ep300b* of teleosts and *ep300* of *L. oculatus*) were calculated using the pearsonr function of scipy.stats module [33].

## Supplementary information

**Additional file 1:** Lineage information of selected species.

**Additional file 2:** Detailed information of selected sequences.

**Additional file 3:** Tblastn search results against RefSeq genomes of 19 fishes.

**Additional file 4:** Maximum likelihood tree of EP300.

**Additional file 5:** Bayesian tree of EP300.

**Additional file 6:** Supplementary information and results.

**Additional file 7:** *ω* ratios of fishes reported by aBSREL test.

**Additional file 8:** Multiple alignment of EP300 sequences and original tree files.

**Additional file 9:** Multiple alignment of four sequences used for structure modeling.

## Data Availability

All data generated or analyzed during this study are included in this published article and its supplementary information files.

## Abbreviations

KAT: Lysine acetyltransferase
EP300: E1A binding protein p300
CREBBP: CREB binding protein, also known as CBP
WGD: Whole genome duplication
MYA: Million years ago
nr: Non-redundant protein sequences
aBSREL: Adaptive branch-site random effects likelihood
ML: Maximum likelihood
CmC: Clade model C
FUBAR: Fast unconstrained Bayesian approximation
MEME: Mixed effects model of evolution
RPKM: Reads per kilobase of transcript, per million mapped reads
