# Supplementary figures and images for: Directional divergence of Ep300 duplicates in teleosts and its implications
Source: BMC Evol Biol. 2020 Oct 31;20:140. doi: 10.1186/s12862-020-01712-6 (PMC7603692; doi:10.1186/s12862-020-01712-6)

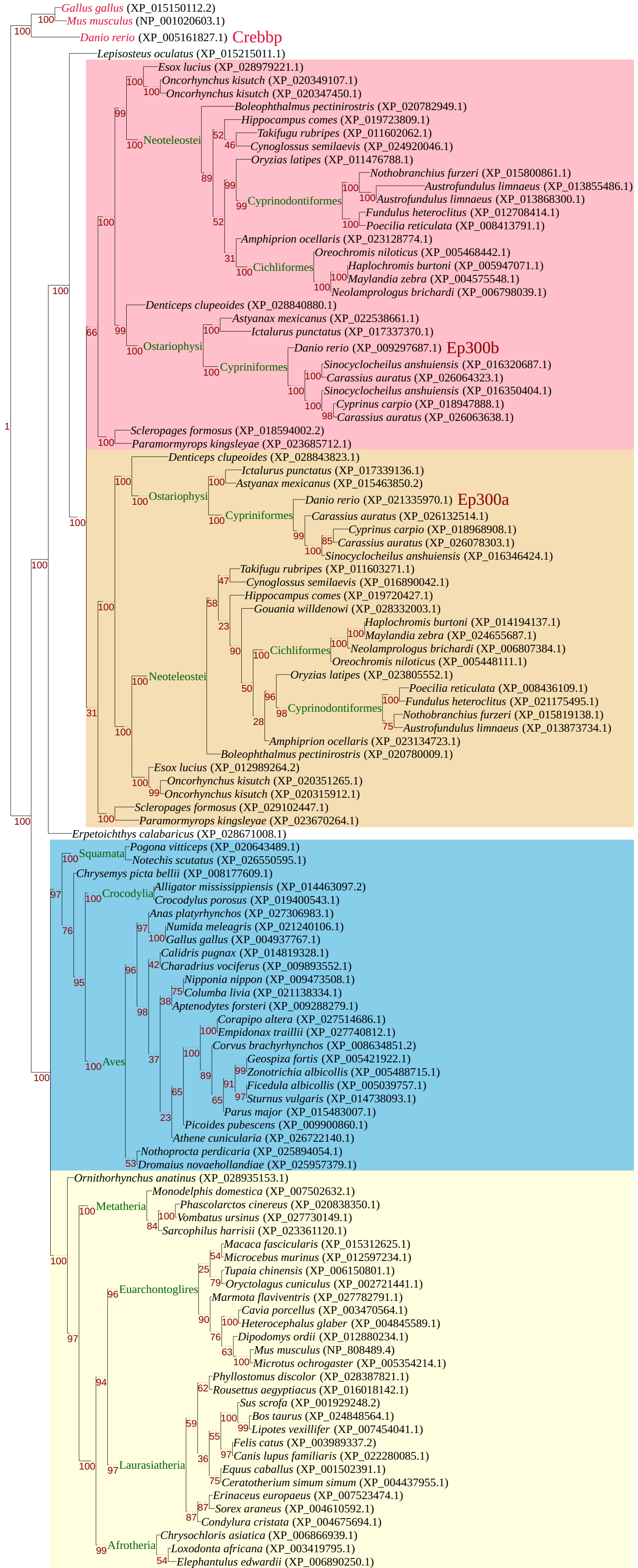

Supplement: Supplementary file 4 — Additional file 4: Maximum likelihood tree of EP300. [file 12862_2020_1712_MOESM4_ESM.pdf]

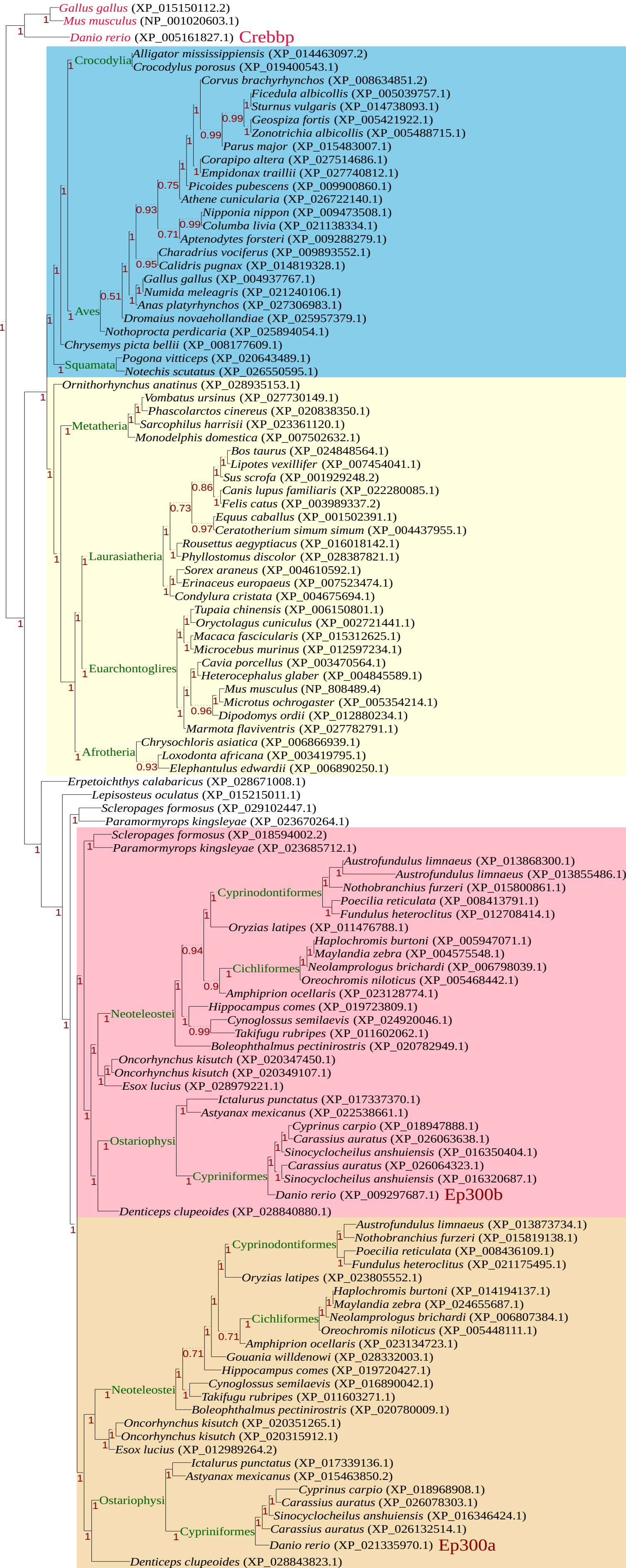

Supplement: Supplementary file 5 — Additional file 5: Bayesian tree of EP300. [file 12862_2020_1712_MOESM5_ESM.pdf]

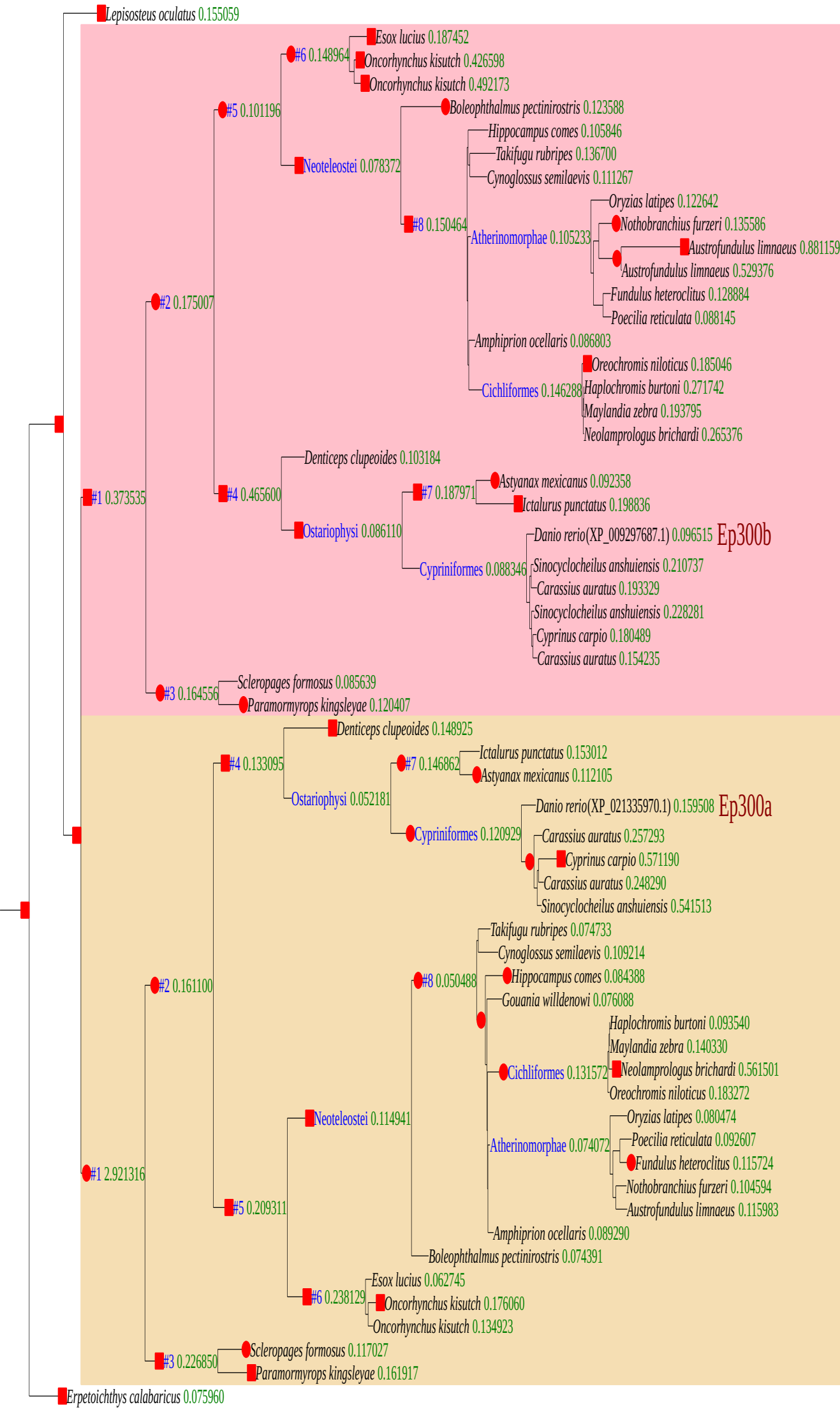

Ep300b

Ep300a

0.13

Supplement: Supplementary file 7 — Additional file 7: ω ratios of fishes reported by aBSREL test. [file 12862_2020_1712_MOESM7_ESM.pdf]
