# Supplementary material for: Directional divergence of Ep300 duplicates in teleosts and its implications
Source: BMC Evol Biol. 2020 Oct 31;20:140. doi: 10.1186/s12862-020-01712-6 (PMC7603692; doi:10.1186/s12862-020-01712-6)
Supplement: Supplementary file 6 — Additional file 6: Supplementary information and results. [file 12862_2020_1712_MOESM6_ESM.docx]

Table S1. Comparison of maximum likelihood tree (RAxML tree) and Bayesian tree

| source | ref | E.size | nRF | RF | maxRF | src-br+ | ref-br+ | subtre+ | treekoD |
| --- | --- | --- | --- | --- | --- | --- | --- | --- | --- |
| Bayesian tree | RAxML tree | 117 | 0.03 | 7.00 | 227.00 | 0.99 | 0.98 | 1 | NA |

The reported values are (from <http://etetoolkit.org/documentation/ete-compare/>):

source target tree used

ref reference tree used to compare with

eff.size Effective tree size used for comparisons (after pruning not shared items)

nRF Normalized Robinson-Foulds distance (RF/maxRF)

RF Robinson-Foulds symmetric distance

maxRF maximum Robinson-Foulds value for this comparison

%src_br frequency of edges in target tree found in the reference (1.00 = 100% of branches are found)

%ref_br frequency of edges in the reference tree found in target (1.00 = 100% of branches are found)

subtrees Number of subtrees used for the comparison (applies only when duplicated items are used to decomposed target trees)

treekoD Average distance among all possible subtrees in the original target trees to the reference tree


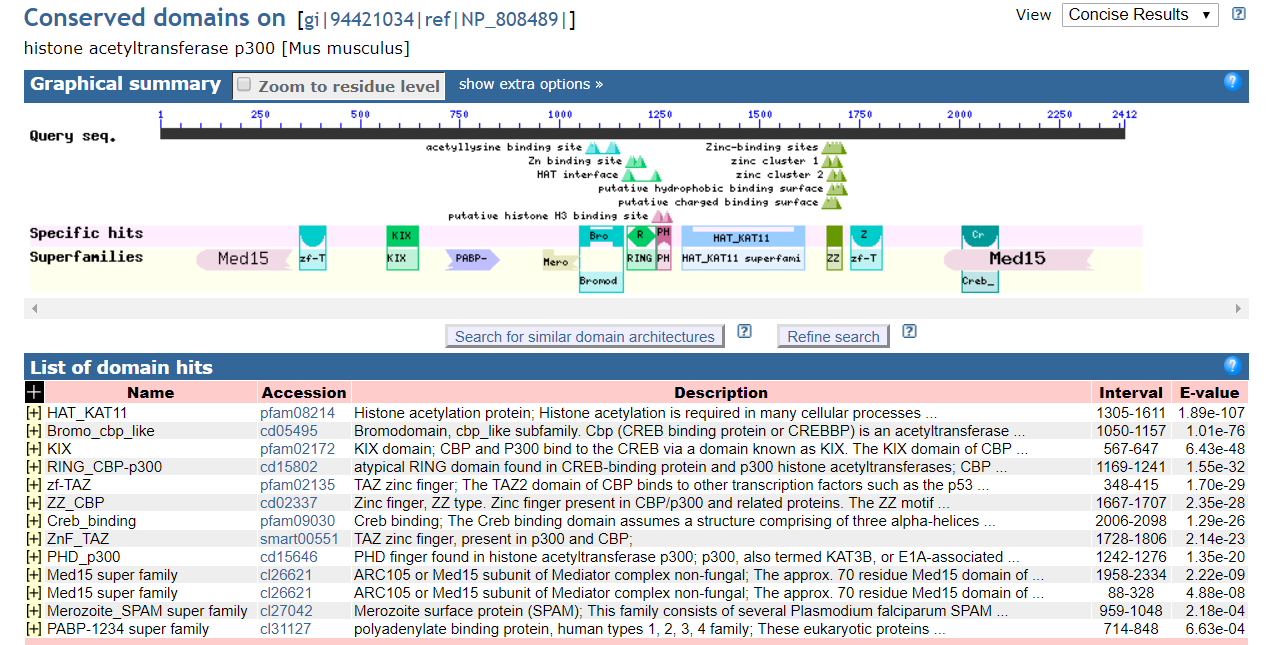


Fig. S1 CDD search result of *M. musculus* EP300.


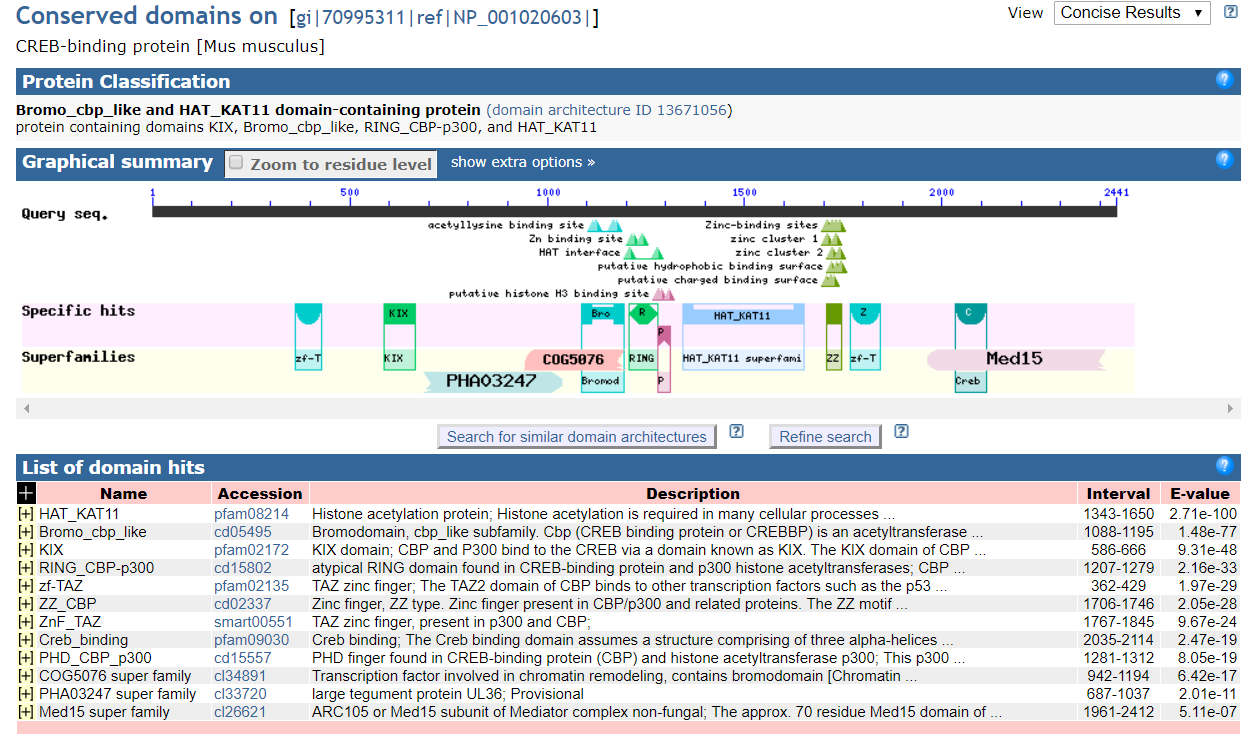


Fig. S2 CDD search result of *M. musculus* CREBBP


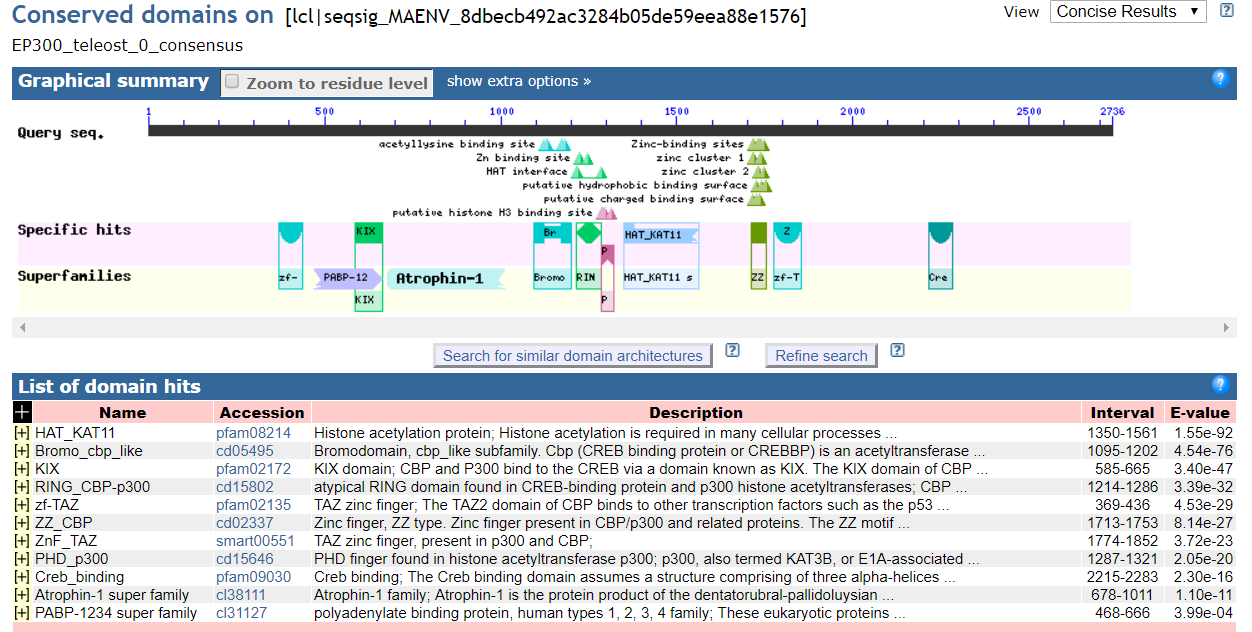


Fig. S3 CDD search result of teleosts’ Ep300a consensus sequence


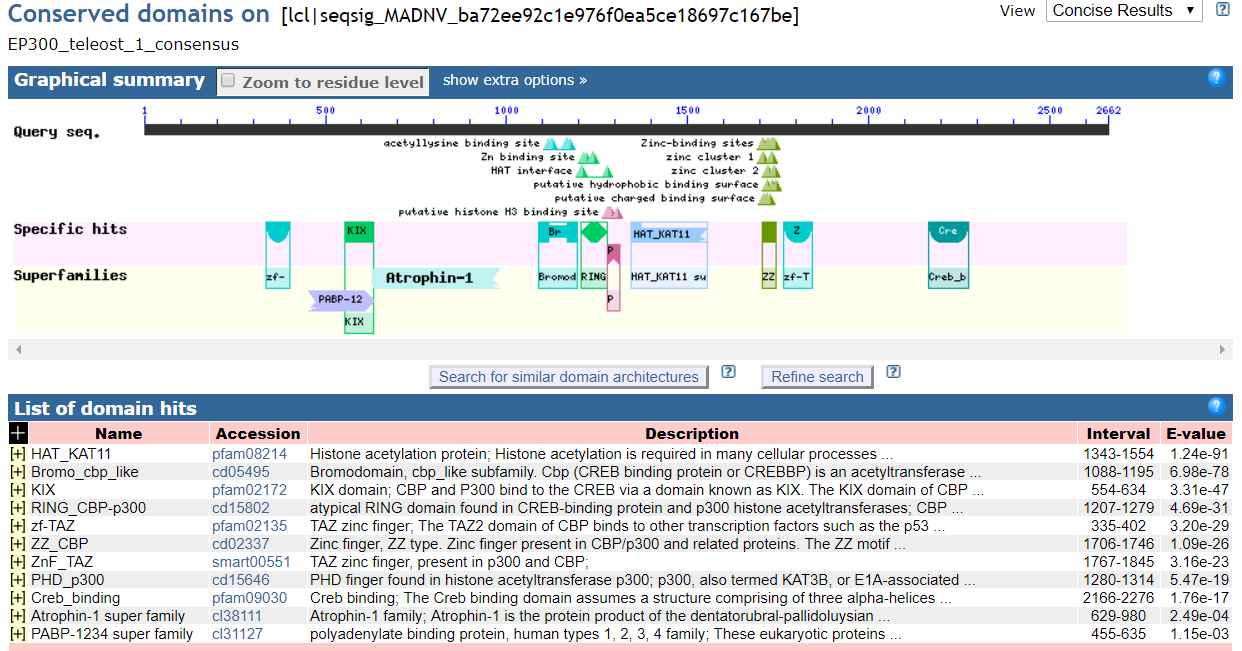


Fig. S4 CDD search result of teleosts’ Ep300b consensus sequence.


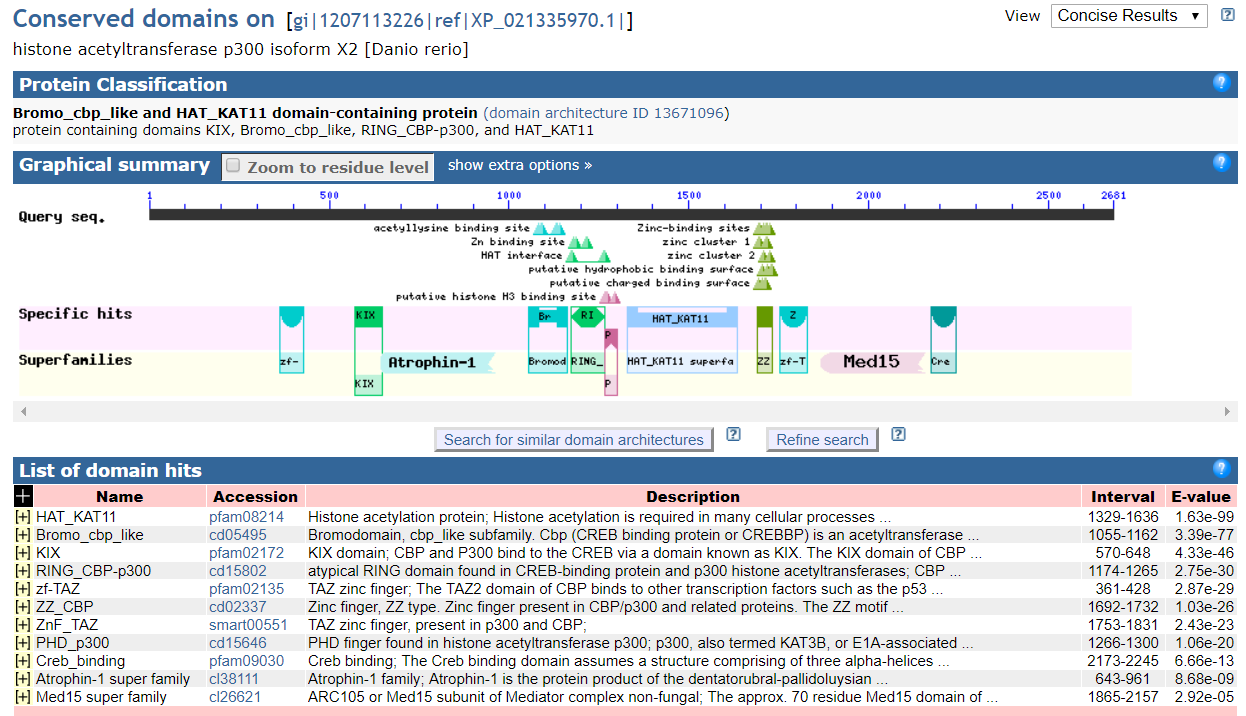


Fig. S5 CDD search result of *D. rerio* Ep300a.


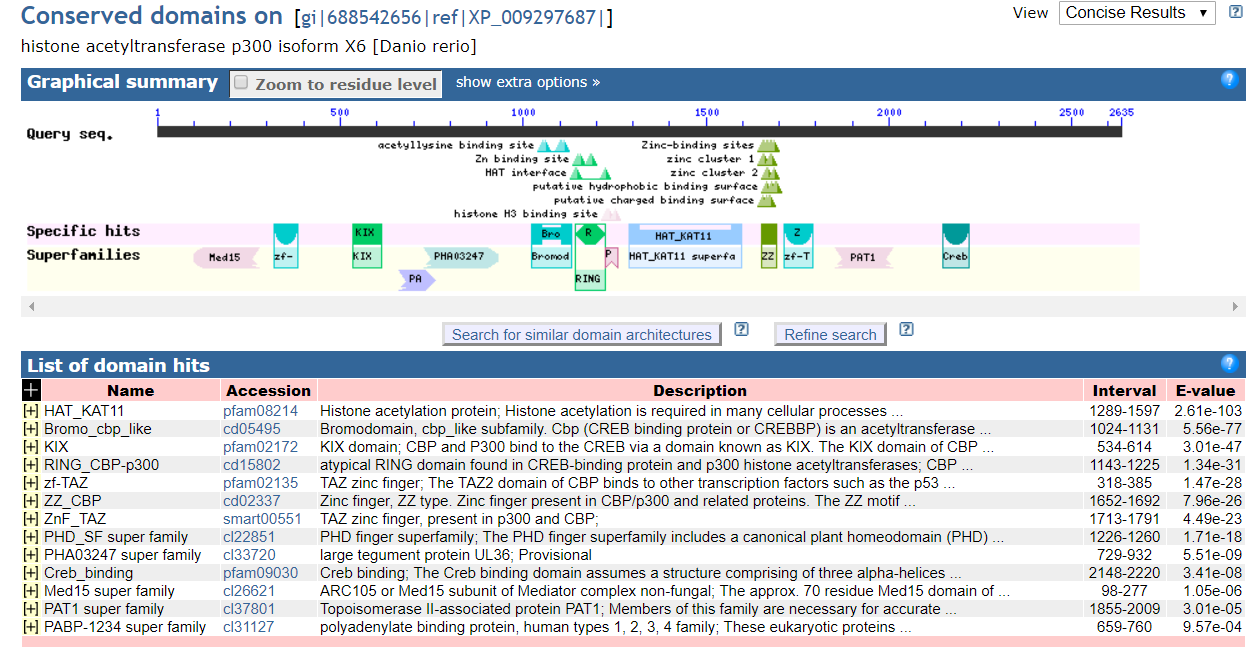


Fig. S6 CDD search result of *D. rerio* Ep300b. Note that in Ep300a there is a specific hit named PHD_p300 while in p300b there is not such a specific hit, but a PHD_SF super family instead. We checked all other isoforms of the Ep300b (gene id 565612) and found that none of them has a PHD_p300 domain either.


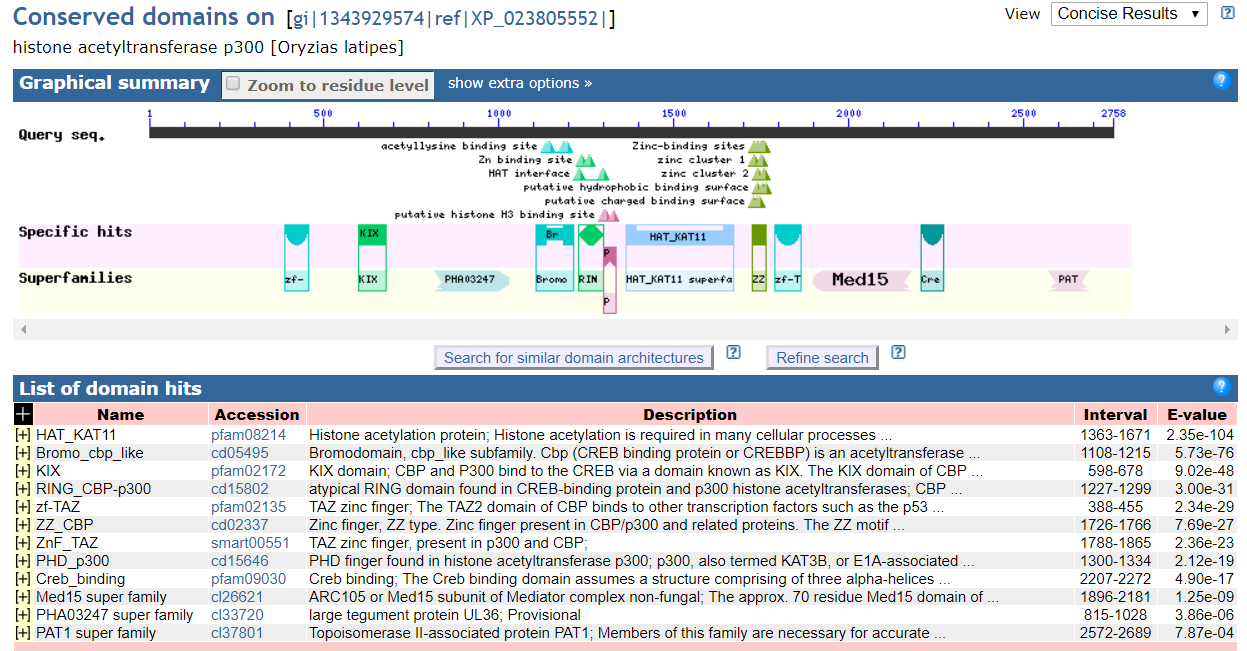


Fig. S7 CDD search result of *O. latipes* Ep300a.


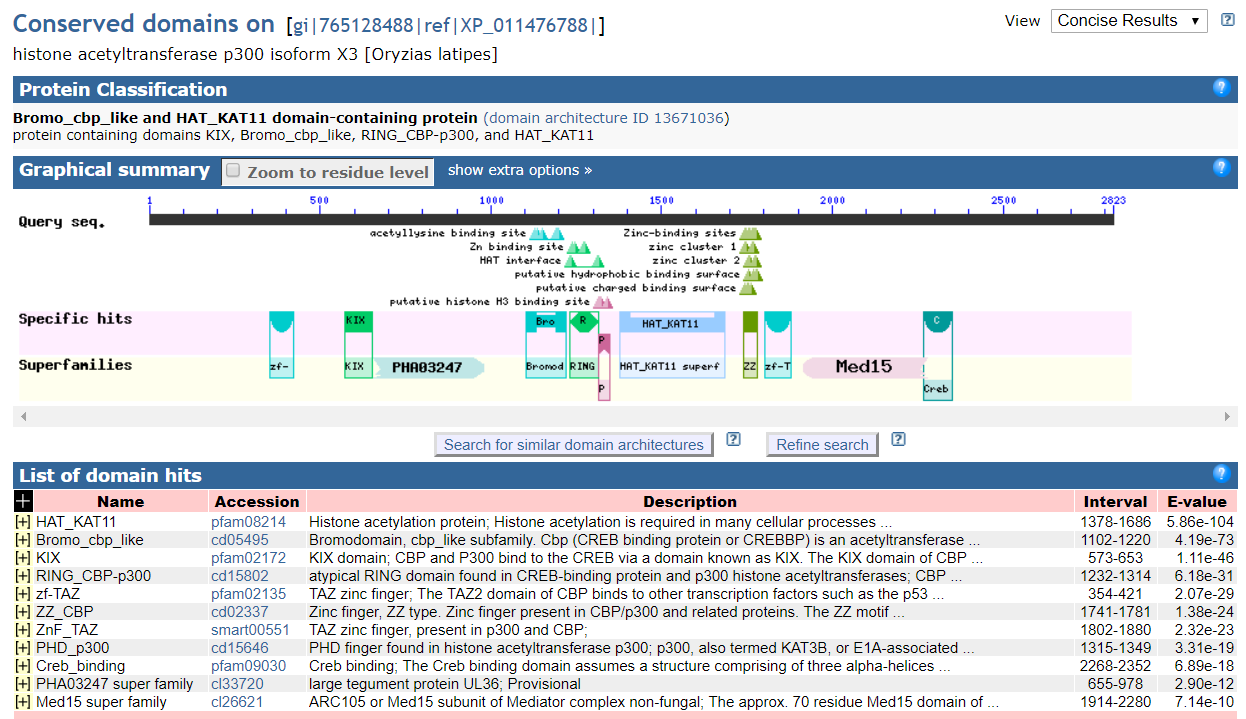


Fig. S8 CDD search result of *O. latipes* Ep300b.

Table S2. Comparisons of proportions of positively selected branches in different clades

| Clade A | positively  selected  branches | total  branches | Clade B | positively  selected  branches | total  branches | *p* value |
| --- | --- | --- | --- | --- | --- | --- |
| Ep300a | 22 | 55 | Ep300b | 21 | 57 | 0.846 |
| Ep300a | 22 | 55 | mammals | 5 | 59 | 0.000117 |
| Ep300a | 22 | 55 | sauropsid | 4 | 49 | 0.000207 |
| mammals | 5 | 59 | sauropsid | 4 | 49 | 1.0 |
| EP300b | 21 | 57 | mammals | 5 | 59 | 0.00029 |
| EP300b | 21 | 57 | squropsid | 4 | 49 | 0.000516 |

Table S3. Estimated accuracy of models of zf-TAZ domain and its flanking regions

| Sequence | Model# | C-score | Estimated | | Clusters (N=11295) | |
| --- | --- | --- | --- | --- | --- | --- |
|  |  |  | TM-score | RMSD | #decoys | density |
| Ep300a of *D. rerio* | model1 | -1.41 | 0.54+-0.15 | 10.6+-4.6 | 2259 | 0.080 |
|  | model2 | -2.45 |  |  | 753 | 0.028 |
|  | model3 | -3.79 |  |  | 370 | 0.007 |
|  | model4 | -4.05 |  |  | 216 | 0.006 |
|  | model5 | -3.74 |  |  | 185 | 0.008 |
| Ep300b of *D. rerio* | model1 | -1.70 | 0.51+-0.15 | 11.4+-4.5 | 2259 | 0.056 |
|  | model2 | -2.46 |  |  | 753 | 0.026 |
|  | model3 | -2.19 |  |  | 753 | 0.034 |
|  | model4 | -4.10 |  |  | 234 | 0.005 |
|  | model5 | -4.22 |  |  | 190 | 0.005 |
| Ep300a of *O. latipes* | model1 | -2.50 | 0.42+-0.14 | 13.7+-4.0 | 1704 | 0.026 |
|  | model2 | -2.53 |  |  | 753 | 0.025 |
|  | model3 | -3.41 |  |  | 733 | 0.011 |
|  | model4 | -3.63 |  |  | 659 | 0.008 |
|  | model5 | -3.42 |  |  | 713 | 0.010 |
| Ep300b of *O. latipes* | model1 | -1.42 | 0.54+-0.15 | 10.7+-4.6 | 3462 | 0.071 |
|  | model2 | -3.35 |  |  | 581 | 0.010 |
|  | model3 | -3.30 |  |  | 554 | 0.011 |
|  | model4 | -3.37 |  |  | 472 | 0.010 |
|  | model5 | -3.73 |  |  | 406 | 0.007 |

C-score is a confidence score of the I-TASSER predictions which is typically in [-5,2]. TM-score and RMSD measure how close the model is to the native structure and both are estimated based on the C-score. TM-score is in [0,1] with a value >0.5 implying the model of correct topology.

Table S4. Estimated accuracy of models of N-terminal side flanking regions of zf-TAZ domain

| Sequence | Model# | C-score | Estimated | | Clusters (N=20200) | |
| --- | --- | --- | --- | --- | --- | --- |
|  |  |  | TM-score | RMSD | #decoys | density |
| Ep300a of *D. rerio*  (1-360 aa) | model1 | -1.52 | 0.53+-1.15 | 10.1+-4.6 | 3041 | 0.062 |
|  | model2 | -2.16 |  |  | 2363 | 0.033 |
|  | model3 | -1.90 |  |  | 2362 | 0.043 |
|  | model4 | -2.86 |  |  | 694 | 0.016 |
|  | model5 | -3.38 |  |  | 647 | 0.010 |
| Ep300b of *D. rerio*  (1-317 aa) | model1 | -2.26 | 0.45+-0.14 | 11.6+-4.5 | 4208 | 0.035 |
|  | model2 | -3.57 |  |  | 797 | 0.009 |
|  | model3 | -2.54 |  |  | 704 | 0.026 |
|  | model4 | -2.04 |  |  | 703 | 0.043 |
|  | model5 | -4.30 |  |  | 595 | 0.004 |
| Ep300a of *O. latipes*  (1-387 aa) | model1 | **-0.31** | **0.67+-0.13** | **7.4+-4.3** | **4725** | **0.158** |
|  | model2 | -1.62 |  |  | 695 | 0.043 |
|  | model3 | -3.80 |  |  | 353 | 0.005 |
|  | model4 | -3.93 |  |  | 316 | 0.004 |
|  | model5 | -4.29 |  |  | 307 | 0.003 |
| Ep300b of *O. latipes*  (1-353 aa) | model1 | **-0.50** | **0.65+-0.13** | **7.6+-4.3** | **12860** | **0.185** |
|  | model2 | -2.07 |  |  | 1464 | 0.038 |
|  | model3 | -2.21 |  |  | 778 | 0.033 |
|  | model4 | -2.77 |  |  | 648 | 0.019 |
|  | model5 | -3.77 |  |  | 642 | 0.007 |


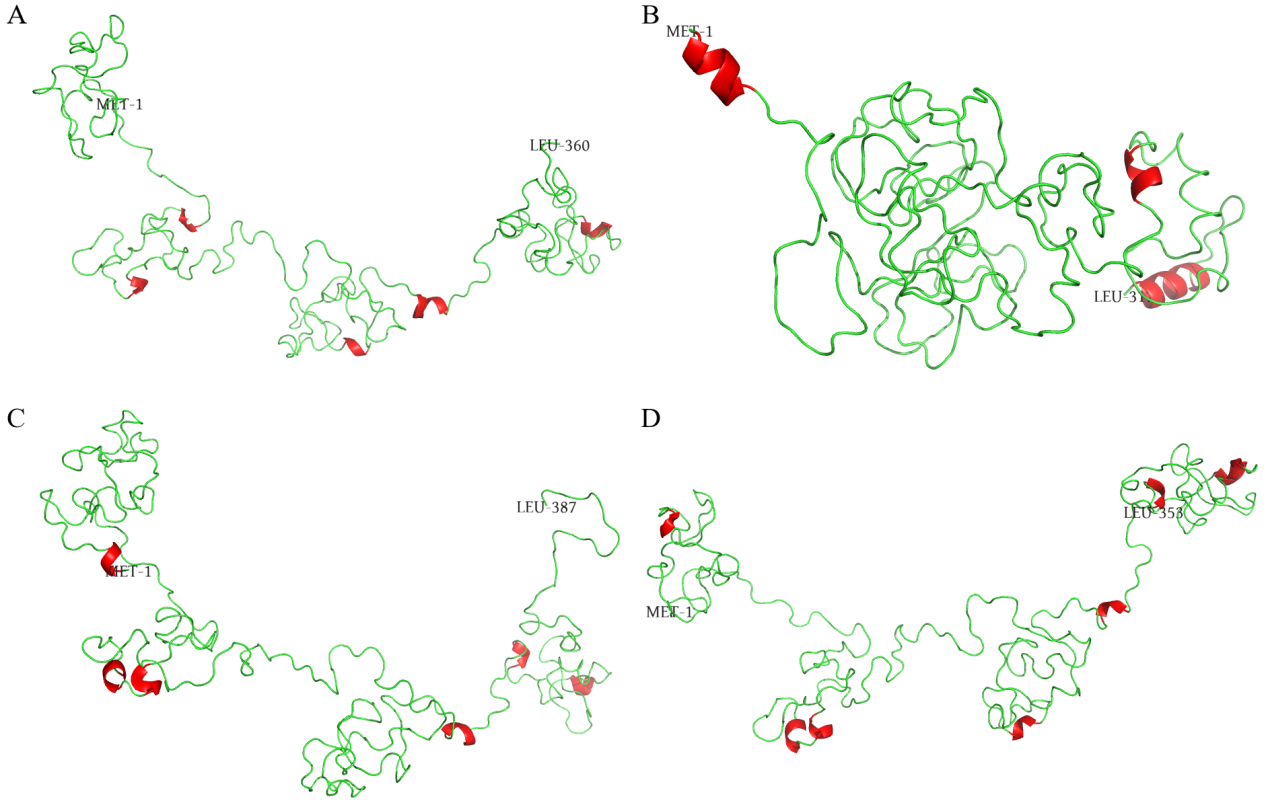


Fig. S9 Structures of N-terminal side flanking region of zf-TAZ domain. The source sequences were Ep300a of *D. rerio* (A), Ep300b of *D. rerio* (B), Ep300a of *O. latipes* (C) and Ep300b of *O. latipes* (D). The first (always MET-1) and last residue of each sequence used for modeling are labeled; α-helixes are colored red. For Ep300a and Ep300b of *D. rerio*, the best models (model 1, as shown in Table S4) were selected. For Ep300a and Ep300b of *O. latipes*, however, the best models are not the best choices: their C-scores and TM-scores were too high to be credible. Actually, model 1 of Ep300a of *O. latipes* contained a number of β-sheets, which are not expected to appear in flanking region of zf-TAZ domain. Therefore, model 2 of Ep300a and Ep300b of *O. latipes* were selected. For Ep300b of *D. rerio*, the α-helixes look longer; however, it should be noted that TM-score of the model 1 was below 0.5.

Table S5. Quality of sequencing data of four fishes

| Species | Number of assembled contigs | Number of bases used for assembly | N50 | N90 | Mean depth | Source link |
| --- | --- | --- | --- | --- | --- | --- |
| *Danio rerio* | 48157 | 135089951 | 3655 | 1488 | 59.79 | <http://phylofish.sigenae.org/ngspipelines/#!/NGSpipelines/Danio%20rerio> |
| *Oryzias latipes* | 42186 | 116128086 | 3654 | 1437 | 47.29 | <http://phylofish.sigenae.org/ngspipelines/#!/NGSpipelines/Oryzias%20latipes> |
| *Esox lucius* | 48567 | 148174033 | 3959 | 1636 | 37.31 | <http://phylofish.sigenae.org/ngspipelines/#!/NGSpipelines/Esox%20lucius> |
| *Lepisosteus oculatus* | 41396 | 124732602 | 4057 | 1602 | 68.5 | <http://phylofish.sigenae.org/ngspipelines/#!/NGSpipelines/Lepisosteus%20oculatus> |

Table S6. Tissue expression profiles of *ep300a/ep300b* based on NCBI SRA study ERP12186

| Organism | Tissue | TPM | | Relative abundance in accordance with PhyloFish data (yes or not) |
| --- | --- | --- | --- | --- |
|  |  | *ep300a* | *ep300b* |  |
| *Danio rerio* | brain | 12.44 | 18.04 | yes |
|  | gill | 8.25 | 11.32 | not |
|  | heart | 4.77 | 2.82 | yes |
|  | liver | 1.66 | 1.54 | yes |
|  | muscle | 2.38 | 1.70 | yes |
|  | eye | 9.11 | 9.04 | - |
|  | gut | 2.62 | 2.66 | - |
|  | skin | 6.70 | 8.21 | - |
| *Oryzias latipes* | brain | 18.48 | 30.99 | yes |
|  | gill | 8.95 | 17.28 | yes |
|  | heart | 2.64 | 1.85 | not |
|  | liver | 2.05 | 2.14 | yes |
|  | muscle | 1.90 | 3.02 | yes |
|  | eye | 10.31 | 15.99 | - |
|  | gut | 5.21 | 6.00 | - |
|  | skin | 3.68 | 6.64 | - |

Methods: Raw RNA-seq reads were downloaded by SRA Run and dumped into fastq format by fasterq-dump in sratoolkit. RefSeq RNA and genome sequences of *D. rerio* and *O. latipes* were downloaded from NCBI FTP site. The links of respective directories are <https://ftp.ncbi.nlm.nih.gov/genomes/refseq/vertebrate_other/Danio_rerio/latest_assembly_versions/GCF_000002035.6_GRCz11/> and <https://ftp.ncbi.nlm.nih.gov/genomes/refseq/vertebrate_other/Oryzias_latipes/latest_assembly_versions/GCF_002234675.1_ASM223467v1/>. RNA and genome sequences were used to build SAF genome index for respective species by salmon index, following tutorials listed on the page <https://github.com/COMBINE-lab/salmon>. Abundances of transcripts of the two species were quantified per tissue by salmon quant. From the gene2accession file (downloaded from <https://ftp.ncbi.nlm.nih.gov/gene/DATA/>) we obtained a full collection of accession numbers of transcript variants of *ep300a/ep300b* genes of the two species. For each gene, TPMs (transcripts per million) of all transcript variants were added to get the gene TPM.
