# Supplementary material for: Directional divergence of Ep300 duplicates in teleosts and its implications
Source: BMC Evol Biol. 2020 Oct 31;20:140. doi: 10.1186/s12862-020-01712-6 (PMC7603692; doi:10.1186/s12862-020-01712-6)
Supplement: Supplementary file 9 — Additional file 9: Multiple alignment of four sequences used for structure modeling. [file 12862_2020_1712_MOESM9_ESM.pdf]

```
                        1       10        20        30        40        50        60
Ep300a_Danio_rerio      MAENVLDSGPPSAKRPKLSSPALSASASDGNDFGSLFDLEHDLPDELISSSELSLANGGDLGQLH
Ep300b_Danio_rerio      MADNVLDSGPPTAKRPKLSSPALSVSASDGNDFGSLFDLEHDLPDELINSSDLGLPNGMDPSQLH
Ep300a_Oryzias_latipes  MAENVLDSGPPSAKRPKLSSPALSVSASDGNDFGSLFDLEHDLPDELISSHEPGLVNGGDLNQLH
Ep300b_Oryzias_latipes  MADNVLESGPPSAKRPKLSSPALSVSASDGNDFGSLFDLEHDLPDELINSSDLGLTNGGDASQLH

                           70                         80        90       100         110
Ep300a_Danio_rerio      TSLGSGGGVIGGA.................VSGGQDAAAKHRQLSELLRHGSTPGAQQ..QGAMG
Ep300b_Danio_rerio      TSLGGGGMS.GPL.................GVSGQDTAAKHKHLSELLRPGAPPSAST....ATG
Ep300a_Oryzias_latipes  TSLGGGAQGLGPAGGNGGAGGIGVGLGPVGAGGGQDAVAKHKQLSELLRSGAPSSNQQGHQGAMG
Ep300b_Oryzias_latipes  TSLGGLGVGIG....................GSGQDAAAKHKQLSELLRAGAP..AQQGGPTSSS

                               120          130       140         150                 160
Ep300a_Danio_rerio      NPGG.ASMGL.FGNMKVSPG..TQSMGPQGQQHLS..MQAGLMQQQ.....QMVDYLN.....RN
Ep300b_Danio_rerio      SPGNAASLGM.MGGLSGGPV..TQGMG..GPQ.....QQPSMMPQP.....GMVGGLN.....RA
Ep300a_Oryzias_latipes  SPGGPTAMGQHLANMKASPGQAPQQMMSQGQQHLSPQQQANMMQQQQNAAAGMMSNMN.....RA
Ep300b_Oryzias_latipes  APSG.PSMAM.MGNINVSSS.GPQGMHPQGQQ.....QQPGLTQQV.....GMVGGVQALSRAAA

                               170       180       190           200       210        220
Ep300a_Danio_rerio      MLGPQKGNGQQQPGGPAPQHQNALASQMMNGSPRIGHH....NPGMGNSNSNLLAEALQQQQQ.Q
Ep300b_Danio_rerio      MMGTQKGNG.........QPQSMMGGQMMNGAPRMGYANVNMNAGMAGN.GNMLPDSLQQPNAGQ
Ep300a_Oryzias_latipes  MMGAQQKANNVQ......QQQGMMGNQVINGSPRMGFG....NQGMPGS.SNLLAETLQQQGA..
Ep300b_Oryzias_latipes  LMGAQNGNTGQ.......QHQGLMGAQVLNGSPRMGFLG...NAGMGTS.GNLLTENLQQQQGGQ

                                 230       240        250       260       270       280
Ep300a_Danio_rerio      TV..GSQGGLRPQQPGAISKMGINAGS.GPYGGPYGQSASQGLGVAGLAPQLQNKPGLPNSPA.Q
Ep300b_Danio_rerio      QM...AQAAMRPQQPGAVNKMGMMGAP.GPYGGSYGQCGGQ....SQLGPQLQNKAGQPNSIN.Q
Ep300a_Oryzias_latipes  ....GGQAAMRGQQPAAMNKMGMMGNPGGPFGGPYGGQSNQALGAAGLGPQLQNKGPMANSLA.Q
Ep300b_Oryzias_latipes  SVGGGVQPGMRPQQPGALNKMSMMANA.GPYGGPYSQSAGQGLAGAGLGPQLQNKAVLPSNVAPQ

                              290       300             310         320       330
Ep300a_Danio_rerio      FNLDKKPLPIHGIPGMASQSS......PVGAGGGGVAA.GMVPNA.QGSLGPGSAGSVVSAAVVG
Ep300b_Danio_rerio      FNMDKKPQLGQNMGGM........................QGPGVVGGVSGPGGTA.........
Ep300a_Oryzias_latipes  FNVDKKSQPMQGMGAMGIQAPQTGVVLPGGPSGAVGGAQGMGPGAPAGLVGPGAQASAASAA..A
Ep300b_Oryzias_latipes  FSMDKKALPGQVMPGMAPQQQQQ....PGGVTGGPIGA........AAAVGGPQVGLAAVGAGPS

                      340       350       360       370       380       390       400
Ep300a_Danio_rerio      GVPPAADPEKRKLIQQQLVLLLHAHKCQRREQANGEVRQCNLPHCRTMKNVLNHMTHCQAGKSCQ
Ep300b_Danio_rerio      .APPAADPEKRKLIQQQLVLLLHAHKCHRREQANGEVRQCNLPHCRTMKNVLNHMTHCQAGKSCQ
Ep300a_Oryzias_latipes  GAPPTADPEKRKLIQQQLVLLLHAHKCQRREQANGEVRQCNLPHCRTMKNVLNHMTHCQAGKSCQ
Ep300b_Oryzias_latipes  TTPPTADPEKRKLIQQQLVLLLHAHKCQRREQANGEVRQCNLPHCRTMKNVLNHMTHCQAGKSCQ

                            410       420       430       440       450        460
Ep300a_Danio_rerio      VAHCASSRQIISHWKNCTRHDCPVCLPLKNAGDKRNQQSESMIATGGVALSSSMG.NVTGGSPSA
Ep300b_Danio_rerio      VAHCASSRQIISHWKNCTRHDCPVCLPLKSAGDKRNQQ..SLLGGAGMAMGGPLGGSLPGGQPSA
Ep300a_Oryzias_latipes  VAHCASSRQIISHWKNCTRHDCPVCLPLKNAGDKRNPQ..SLLGAAGAGLGSSLG.AVPGAQPST
Ep300b_Oryzias_latipes  VAHCASSRQIISHWKNCTRHDCPVCLPLKNAGDKRNQQ..SLLNSAAMGLVNALGSGVPVGQSST

                        470       480       490       500
Ep300a_Danio_rerio      PSLNTPGQIDPSSIERAYAALGLTYQGNQAPPQ................................
Ep300b_Danio_rerio      PNINPPSQIDPSSIERAYAALGLTYQGNQASNQNQQTSVP.....GQPGMRSLNNMAGGNSMGVN
Ep300a_Oryzias_latipes  PNLNPTSQIDPSSIERAYAALGLTYQGNQIQTQSSQPN......QGPAGMRPLNPI.GANPMGVN
Ep300b_Oryzias_latipes  PNLNPTNQIDPSSIERGYAALGLTYQGNQMPQQLPQTNMPNQGLQGQPGMRTLNTM.GGNPM...

Ep300a_Danio_rerio      .................
Ep300b_Danio_rerio      GGVGAPMSNQHPGMLPD
Ep300a_Oryzias_latipes  .................
Ep300b_Oryzias_latipes  .................
```
